# Transcriptome profiling reveals exposure to predicted end-of-century ocean acidification as a stealth stressor for Atlantic cod larvae

**DOI:** 10.1038/s41598-019-52628-1

**Published:** 2019-11-15

**Authors:** F. H. Mittermayer, M. H. Stiasny, C. Clemmesen, T. Bayer, V. Puvanendran, M. Chierici, S. Jentoft, T. B. H. Reusch

**Affiliations:** 1 GEOMAR Helmholtz Centre for Ocean Research Kiel, Marine Evolutionary Ecology, Düsternbrooker Weg 20, 24105 Kiel, Germany; 2 Dept. of Economics, Kiel University, Sustainable Fisheries, Wilhelm-Seelig-Platz 1, 24118 Kiel, Germany; 3 Nofima AS, Muninbakken 9, NO-9019 Tromsø, Norway; 4 Institute for Marine Research, Framsenteret, Hjalmar Johansens gate 14, NO-9007 Tromsø, Norway; 5 Centre for Ecological and Evolutionary Synthesis (CEES), Department of Biosciences, University of Oslo, Postboks 1066, NO-0316 Oslo, Norway

**Keywords:** Climate-change ecology, Molecular ecology

## Abstract

Ocean acidification (OA), a direct consequence of increasing atmospheric CO_2_ concentration dissolving in ocean waters, is impacting many fish species. Little is known about the molecular mechanisms underlying the observed physiological impacts in fish. We used RNAseq to characterize the transcriptome of 3 different larval stages of Atlantic cod (*Gadus morhua*) exposed to simulated OA at levels (1179 µatm CO_2_) representing end-of-century predictions compared to controls (503 µatm CO_2_), which were shown to induce tissue damage and elevated mortality in *G. morhua*. Only few genes were differentially expressed in 6 and 13 days-post-hatching (dph) (3 and 16 genes, respectively), during a period when maximal mortality as a response to elevated *p*CO_2_ occurred. At 36 dph, 1413 genes were differentially expressed, most likely caused by developmental asynchrony between the treatment groups, with individuals under OA growing faster. A target gene analysis revealed only few genes of the universal and well-defined cellular stress response to be differentially expressed. We thus suggest that predicted ocean acidification levels constitute a “stealth stress” for early Atlantic cod larvae, with a rapid breakdown of cellular homeostasis leading to organismal death that was missed even with an 8-fold replication implemented in this study.

## Introduction

Global change, caused by diverse anthropogenic activities, is the defining characteristic of the Anthropocene^1^ and has already started to affect marine ecosystems (reviewed by Doney *et al*.^2^). One of the major first order effects with direct causal linkage to human activity is ocean acidification (OA). It is caused by the uptake of rising atmospheric CO_2_ concentrations, from fossil fuel burning and altered land use, by ocean waters. The increases in ocean *p*CO_2_ and the resulting decreased ocean pH as well as lowered carbonate saturation state^3^ have been shown to impact marine ecosystems^3–5^, particularly calcifying species including foundation species such as corals^6^. For marine fish species, results are more complex, i.e. change in thermal window range for different life stages^7^. Most adult fish seem to be less susceptible to increased *p*CO_2_ compared to early life stages^8–10^. Due to their high capability of acid-base regulation some adult fish can tolerate *p*CO_2_ levels of >8000 µatm^11^, far exceeding the projections of ocean acidification for the near future^12^. In contrast, exposure of early life stages to increased *p*CO_2_ has been shown to induce severe effects on their performance such as decreased hatching success^10^, increased larval mortality^9^, but also increase in growth and faster developmental patterns^8,13,14^, decreased oxygen consumption rate^15^ and impaired sensory abilities and behaviour^16,17^. Further, changed otolith and bone development^14,18,19^ as well as tissue and developmental damages^14,20,21^ have been observed. Yet, it has so far been difficult to link all these effects to fitness consequences.

Some regulatory mechanisms and capabilities of adult fish to achieve and sustain acid-base balance such as bicarbonate regulation in the blood plasma or hydrogen and bicarbonate excretion have been identified^22–24^, but very little is known about acid-base regulation in early life stages. Gill epithelia, the most important tissue for acid-base regulation in adult fish^22^, are only developing in larvae, with a complete absence at hatch in cod larvae (for cod see^25^). During early development, all proton excretion occurs via chloride cells (Na^+^/K^+^ ATPase-rich ionocytes). These are located in the skin^26^ and the primordial gill cavity^10^. The detrimental effects of OA on larvae are most likely due to the organism’s limited ability to regulate their acid-base balance or the cost of increased regulation. The increased cost of acid-base regulation in an acidified ocean could have potential impacts on the fitness of the larvae^14,24,27^.

With the increasing availability of next generation sequencing techniques, global transcriptome profiling in non-model organisms has become possible and affordable. This allows to elucidate the molecular basis for observed physiological impairments, and thus, a more detailed characterization of the stress phenotypes^28^, with the ultimate goal to understand how exactly OA perturbs larval physiology. There are, so far, few attempts on untangling the full transcriptomic response to ocean acidification stress in fish. Most studies are based on candidate gene approaches concentrating on acid-base regulation^29,30^ in adult fish. More recent studies have, however, employed whole transcriptome sequencing methods such as RNAseq (for a review see^31^) and identified differentially expressed (DE) genes. In these studies the focus was mainly on the cellular stress response^32^ and neurological signalling in adult fish^33^. Underlying transcriptomic mechanisms of above mentioned effects during early development remain unclear, and thus, even fewer studies have focused on the gene expression changes to ocean acidification in eggs and larvae^13^.

Ocean acidification, including a decrease in pH, as well as changes in temperature and hypoxia, can impact the function of many cellular processes and cause damages to cellular macromolecules such as DNA and proteins^34^. In order to avert damage, cells can react to harmful changes in their cellular environment by means of the cellular stress response (CSR) defined by Kültz^34^. The CSR is manifested as the minimal stress proteome, consisting of circa 300 proteins with a core number of highly conserved genes throughout all organisms^35^. Proteins of the CSR are mainly involved in molecular chaperoning, DNA repair, protein folding, redox regulation and energy metabolism. Cells initiate a CSR in response to damaged macromolecules by increasing transcription of the genes encoding for the stress proteome. This leads to increased concentration of CSR proteins while their activity is further controlled by post translational modifications^35^. Increased *p*CO_2_ levels are known to induce differential expression of genes of the CSR in tissues of adult fish^32^ but again very little is known about the CSR in larval fish under the stress that is imposed by increased *p*CO_2_.

In order to elucidate the physiological basis of ocean acidification effects in Atlantic cod (*Gadus morhua*) larvae, a key species of ecological and economic importance in the North Atlantic, in response to simulated ocean acidification levels, this study employed global gene expression profiling via mRNA sequencing (RNA-Seq). Atlantic cod embryos and subsequent larvae were exposed to either ambient/control (503 µatm) or end-of-century *p*CO_2_ concentrations (1197 µatm). The sampling dates for the RNA-Seq were based on important physiological changes occurring in the cod larvae^36,37^ and on the observation of phenotypic traits and mortality^9,14^ at those time points. The phenotypic effects of that experiment have been reported before^8,13^. The *p*CO_2_ treatment concentrations reflecting future ocean states were chosen according to RCP 8.5^12^ and represent the global mean ocean acidification level predicted for 2100. Note that on a regional scale (the Barents Sea) these levels will most likely be reached earlier or under more optimistic climate scenarios^38^. A more detailed description of the experimental set-up has been published elsewhere^8^. In short, end-of-century prediction OA levels were shown to induce dramatic consequences for fitness related measurements, i.e. a doubling in the daily mortality rate (from 7% in ambient to 13% in high *p*CO_2_ treatment)^9^ and other severe phenotypic differences like changes in ossification rates in the vertebrae, gill development and tissue histology such as liver vacuolization^14^.

Although a small number of studies have examined gene expression in fish larvae in response to ocean acidification, these studies have been limited to candidate genes related to stress response^39^ or acid-base regulation^13^, which did not allow for the identification of potentially important genes and pathways under differential expression when exposed to simulated ocean acidification. To our knowledge, this is the first study that addresses the effect of OA on the whole transcriptome of marine fish larvae comparing different developmental stages. In addition, special emphasis was placed on gene families connected to the CSR to address the question if Atlantic cod during early developmental stages show signs of cellular stress in response to ocean acidification.

## Results

### Larval dry weight

**Figure 1:**
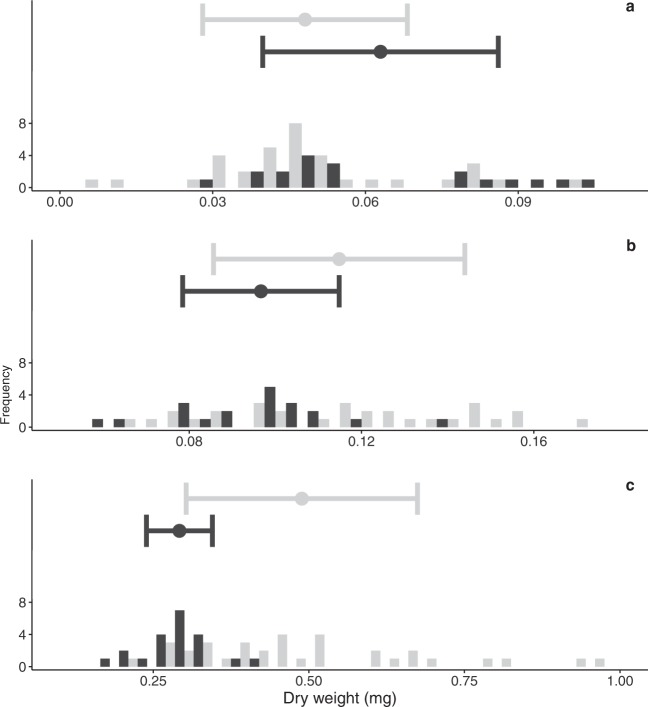
Means and histograms of larval dry weight in mg at (**a**) 5 dph, (**b**) 15 dph and (**c**) 36 dph (modified from Stiasny *et al*.^13^). Grey bars correspond to larvae from the high *p*CO_2_ (~1179 μatm) treatment, black bars correspond to larvae from the ambient (~503 μatm) treatment. The centre represents the mean, the whiskers standard deviation (SD).

To assess the impact of simulated ocean acidification levels on growth performance in the Atlantic cod larvae, dry weight measurements were recorded at 5, 15 and 36 days-post-hatch (dph). The first two early larval stages measured: 5 dph (Fig. 1a) and 15 dph (Fig. 1b), showed no differences in dry weight between ambient and high *p*CO_2_ treatment (F_1,3.73_ = 6.00, *p* = 0.08 and F_1,3_ = 2.06, *p* = 0.25, respectively) (full results SI Table 1). A significant difference in dry weight was observed at 36 dph (Fig. 1c), where larvae from the high *p*CO_2_ treatment were significantly heavier than larvae from the ambient treatment^14^. With increasing larval age the variance in dry weight of larvae from the high *p*CO_2_ treatment increased compared to the variance in the ambient treatment.

### Global gene expression profiling via RNA sequencing

For assessment of changes in individual gene expression patterns, transcriptome profiling (RNAseq) was performed on 8 biological replicates for each treatment (ambient and increased *p*CO_2_) at each of the three sampling points 6, 13 and 36 dph. Illumina sequencing generated a total of 3.4 * 10^9^ raw reads (sample average 35.5 * 10^7^ read-pairs 151 bp in length) with a range of 3.07–6.61 * 10^7^ reads. After adaptor trimming and discarding unpaired reads, an average of 3.52 * 10^7^ paired reads per biological replicate remained (For more information SI Table 2). On average, 69.2% of reads per sample could be uniquely assigned to the transcriptome generated from the annotated genome.

**Figure 2:**
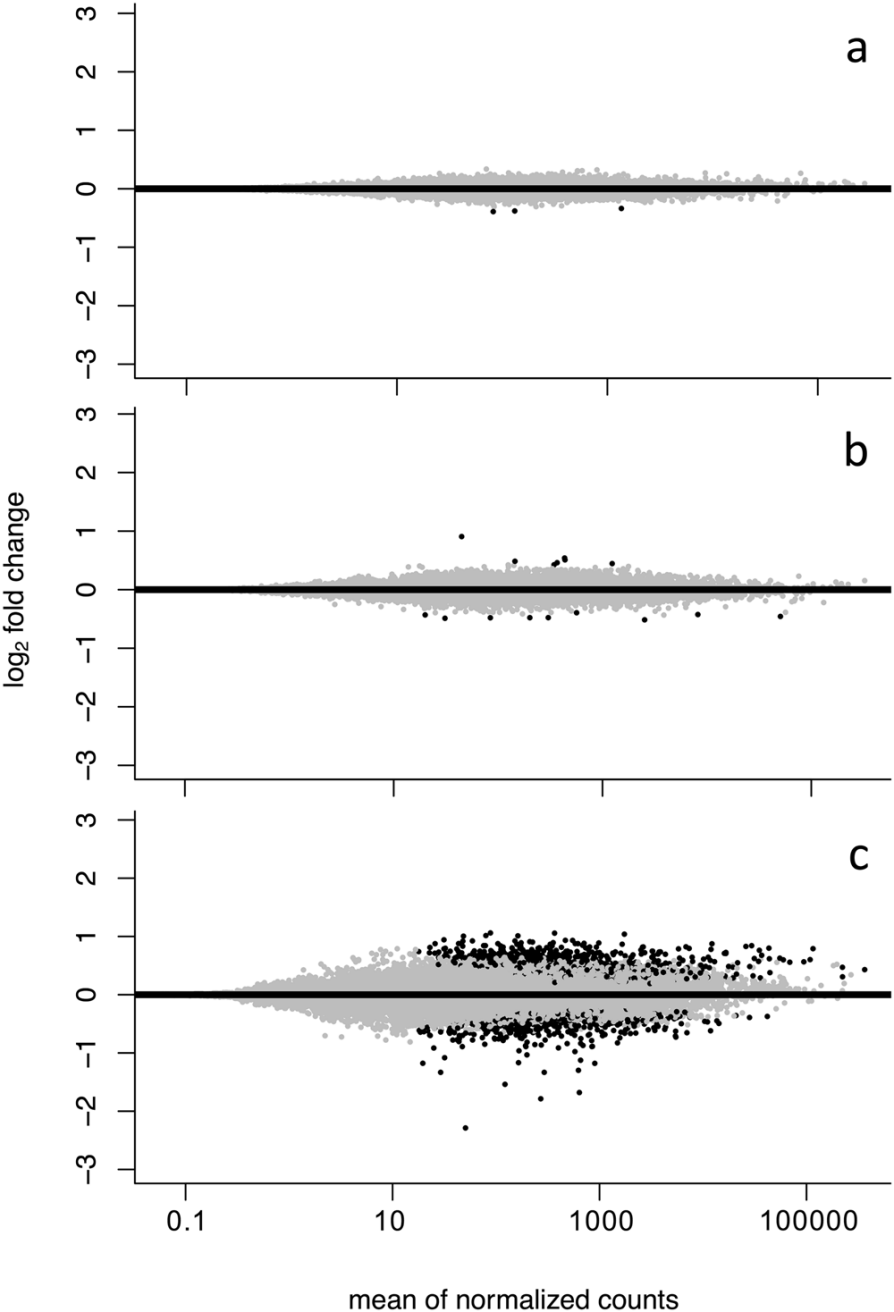
Plots of log_2_ fold change against mean of normalized counts (MA) for (**a**) 6 dph (**b**) 13 dph and (**c**) 36 dph larvae. Black dots represent genes significantly differential expressed (p_adj_ < 0.05, false discovery rate correction (FDR) = Benjamini-Hochberg(BH)) between ambient (~503 μatm) and high (~1179 μatm) *p*CO_2_ treatment. Grey represents no significant differential expression between treatments.

Larvae sampled at 6 dph revealed only three differentially expressed (DE) genes (p_adj_ < 0.05, FDR = BH) (SI Table 4). All three genes were downregulated in the high *p*CO_2_ treatment compared to the ambient treatment. In 13 dph larvae, 16 genes were differentially expressed but unlike the youngest larvae, 7 genes were upregulated under high *p*CO_2_ (SI Table 5). This low transcriptomic response at the first two early larval stages stood in strong contrast to the response manifested in the expression patterns of the oldest group as visualized in the fold change/mean counts plots (MAplots) (Fig. 2). In 36 dph larvae, 1413 genes were differentially expressed, 19 of which were up- or down-regulated by a factor of 2 or more (SI Table 6).

**Figure 3:**
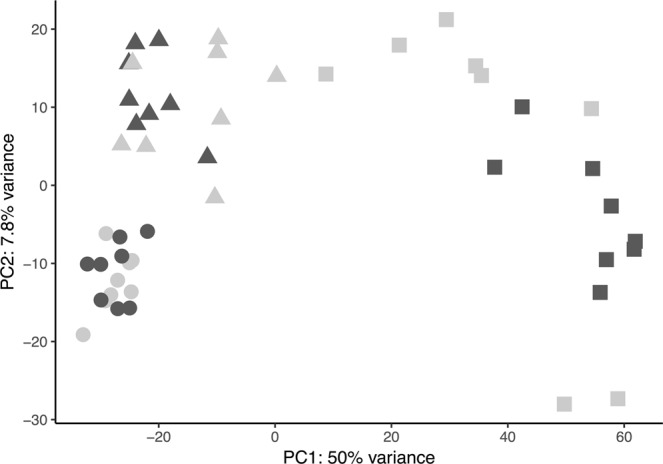
Principle component analysis of total gene expression profile, visualized for first and second component. Shapes represent larval age groups, circle 6 dph, triangle 13 dph and, squares 36 dph, while colours correspond to larval treatment, black ambient (~503 μatm) and grey high (~1179 μatm) *p*CO_2_.

This pattern of gene expression between the treatments at different ages was visualized in a principal component plot (Fig. 3, PC1 = 50%, PC2 = 7.8%). All three age groups were well separated by the 1^st^ and 2^nd^ component of the PCA representing differences in gene expression patterns between age groups (ANOSIM 999 permutations, R = 0.799, *p* = 0.001). For young larvae (6 and 13 dph), replicates did not segregate according to *p*CO_2_ treatment, indicating only small differences in transcriptomic response to ocean acidification. On 36 dph, replicates were partially clustered by treatment, yet showed a large spread along both principal component axis, reflecting the large number of DE genes. Further, none of our pre-defined candidate genes, both in the CSR or acid-base regulation were identified among the 25 main contributing genes to loadings in the first and second principle component (SI Table 3a,b).

### Gene ontology analysis

A gene ontology enrichment analysis was performed on the 1413 differentially expressed genes in 36 dph cod larvae and yielded two significantly enriched GO terms (*p*_adj._ < 0.05, FDR = BH): extracellular matrix (GO:0031012) and mitotic chromosome condensation (GO:0007076). GO terms related to the CSR such as “response to stress” GO:0006950 and its child terms including “cellular response to stress” GO:0033554 were not enriched. Further, no GO terms related to acid-base regulation, for example “proton transport” GO: 0015992 or “bicarbonate transmembrane transporter activity” GO:0015106, were significantly enriched.

### Assessment of candidate genes of the cellular stress response (CSR)

At both early time points, none of the genes belonging to the cellular stress response (CSR) (see Table 1 in^35^) were differentially expressed at 6 or 13 dph. None of the differentially expressed genes could be associated to cell or protein damage. At 36 dph, of 45 gene families in the highly conserved CSR, five families and/or genes were not present in the available annotations or were not expressed in the samples. This leads to a total of 185 annotations related to the CSR of which 14 were differentially expressed (p_*adj*_ (FDR = BH) ≤ 0.05) (7.6%). Genes coding for HSP60 chaperonin, peroxiredoxin, superoxide dismutase, thioredoxin, RAD51 DNA repair proteins, Lon protease, Long-chain- fatty acid ABC transporters were expressed in both treatments with no significant differences.

Most differentially expressed genes showed low fold changes (l2FC between −0.54 and 0.85), including a number of well-known stress-related genes belonging to the universal CSR. Heat shock protein 70 kDa (HSP70) was present in several variants in the reference transcriptome, one of which was upregulated by a fold change of 1.68 (l2FC = 0.75, p < 0.05). In addition, Serpin, also known as heat shock protein 47 kDa (HSP47), was moderately upregulated in the high *p*CO_2_ treatment (l2FC = 0.72, p < 0.01). Additionally, a number of DNA J homologs (Dnajc9 and Dnajb6-b) were differentially expressed (l2FC = 0.53 and 0.61, p < 0.05 and p < 0.05, respectively). Upregulated genes related to the CSR further include: one of two homologs of Seratin proteases HTRA3 and HTRA1A (l2FC = 0.63, p < 0.05 and l2FC = 0.49, p < 0.01 respectively), Long-chain-fatty-acid CoA ligase 6 ACSL6 (l2FC = 0.5, p < 0.05), Beta-enolase ENO3 (l2FC = 0.51, p < 0.05), Propyl endopeptidase Prep (l2FC = 0.34, p < 0.05), MutS/MSH protein homolog 4 MSH4 (l2FC = 0.38, p < 0.05), Aldo-keto reductase AKR1B10 (l2FC = 0.23, p < 0.05), Aldehyde dehydrogenase ALDH1A3 (l2FC = 0.39, p < 0.05), Nucleoside diphosphate kinase NME1-2 (l2FC = 0.60, p < 0.01) and Glycerol-3-phosphate dehydrogenase gpd1 (l2FC = 0.85, p < 0.01).

### Candidate gene analysis for acid-base and osmoregulation

At both early sampling dates, none of the pre-defined candidate genes encoding important enzyme components of acid base regulation, for example Na^+^/H^+^ exchanger, HCO_3_^−^ transporters and Carbonic anhydrase among others^23,29^ were differentially expressed. Later, at 36 dph, of 41 annotations related to acid-base regulation only one (CA4) was differentially expressed between the treatments. This one of three gene models encoding Carbonic anhydrase 4 (CA4) was downregulated in the high *p*CO2 treatment (l2FC = −0.54, p < 0.01). Further, a number of ammonium transport rhesus (Rh) proteins, involved in CO_2_ transport across membranes, were differentially expressed. One ammonium transporter, namely Rh type C2 (rhgc2) was down regulated (l2FC = −0.37, p < 0.05) while one of two annotations of ammonium transporter Rh type B (rhbg) was up regulated (l2FC = −0.40, p < 0.05) in the high *p*CO_2_ treatment. Only one of the genes directly involved in acid-base regulation, Pendrin (SLC26a4), was not identified in the reference genome. Of the ammonium transport Rh proteins, two out of seven were expressed at significantly different levels. Further none of the candidate genes are present in the 25 main contributing genes to the first (SI Table 3a) and second principle component (SI Table 3b) in Fig. 3.

## Discussion

In this study we correlated previously measured strong phenotypic effects in response to realistic end-of-century^9,14^ with global gene expression patterns in Atlantic cod larvae at a high level of replication (N = 8) and sequencing coverage. We compared different developmental stages and therefore possible changes in regulatory pathways in experimental fish that showed a doubling in the mortality rates, histological damage and changes in ossification patterns when exposed to high *p*CO_2_ levels.

The fold changes observed in our study were very modest, but we note here, that even small fold changes may contribute significantly to cellular metabolism and vice versa owing to post-translational processes^40^.The results were surprising in that we found little gene expression differences during the early phase of the experiment when most of the mortality occurred (6 dph and 13 dph)^9^. This is in strong contrast to the observed responses in fitness related measurements in cod larvae in the same experiment, such as growth and survival^9,14^.

Once the larvae were further developed (36 dph), many more differences in gene expression between the treatments were observed, namely 1413 DE genes. The only two categories that were significant in a GO enrichment analysis, “pathways of extracellular matrix” and “mitotic chromosome condensation” suggest that the observed gene expression differences in 36 dph old larvae were driven by divergent rates of larval development rather than sensitivity to CO_2_ per se. This is in line with previous studies showing enhanced developmental rates in response to increase in *p*CO_2_ levels, but so far the mechanisms are unknown^13,19^.

Additional support for faster development under ocean acidification was provided by a group of DE genes that are expressed in highly variable levels along the developmental stages of *Danio rerio*^41,42^. Protein fosB, Krueppel-like factor 4, Cyclicl AMP-dependent transcription factor ATF-3, Pleckstrin homology like domain family A member 2, Amphiregulin and Myosin heavy chain (fast skeletal muscle) (SI Table 5) are all expressed at variable levels along the larval development in zebra fish^41,42^ and can be assumed to follow similar patterns in other teleosts. However, it is unclear whether the developmental changes are caused by direct effects of OA on growth and ossification or if the observed differences are due to the strong mortality observed in the early ages^9^ potentially selecting for fast growth or combination of both with related trade-offs^14^.

We then assessed whether larvae showed transcriptomic signs of the well-defined general cellular stress response (CSR) at the late sampling date. 14 gene candidates belonging to 10 gene families were DE in 36 dph larvae, which is less than 8% of all possible candidates (total 185) of the CSR present at that sampling point.

**Figure 4:**
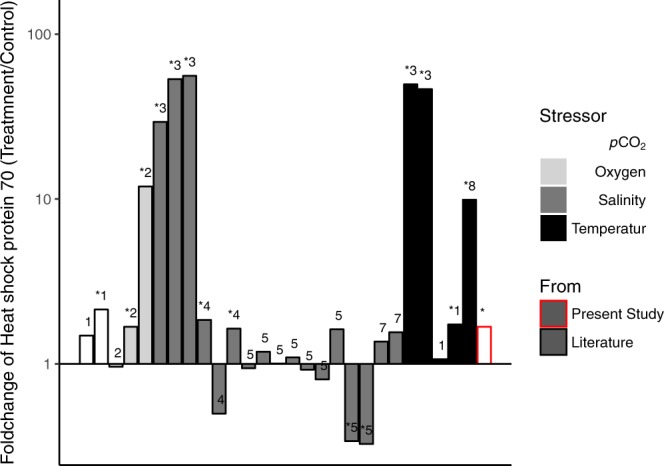
Available data from literature of heat shock protein 70 (HSP70) fold changes in fish in response to chronic exposure to abiotic environmental stressors, * marks significant differences between treatments, numbers show reference numbers in SI Table 8. For further information see SI Table 8.

While we found HSP 70, which is a universal indicator of the stress response, to be differentially expressed, fold changes observed were modest (+1.68) and much lower compared to published data assessing stress effects on fish such as salinity, oxygen and temperature (Fig. 4 and SI Table 8), where fold changes ranged from 2.5 to 372 FC^43–45^. Interestingly, it seems that the reaction in tissues of adult fish generally results in much higher fold changes, e.g. to temperature in rainbow fish^44^ and salinity in sea bass^46^.

We then compared our results to previous studies that identified a variable stress response in studied larval fish exposed to increased *p*CO_2_, all of which were target gene studies using Q-PCR or protein assays. When investigating the expression patterns of HSP70 in whole fry (8 days post fertilization) of big head carp (*Hypophthalmichthys nobilis*) and silver carp (*H. molitrix*) in response to extreme *p*CO_2_ levels (43000 µtam) in freshwater, only larvae from the latter species showed an upregulation in HSP70^39^. Nevertheless, such *p*CO_2_ levels are not possible in most parts of the oceans. Further, the stress response to predicted future *p*CO_2_ levels was assessed in the larvae of a tropical flatfish^47^. Protein levels of HSP70 in whole Senegalese sole (*Solea senegalensis*) larvae incubated and reared in ambient and high *p*CO_2_ (~1600 μatm) levels differed only in 30 day old larvae^47^. They were significantly higher in the ocean acidification treatment compared to the control, while protein levels did not differ in 10 dph larvae. These results are in line with our study, indicating that only later larval stages display a very limited cellular stress response to increased *p*CO_2_ compared to earlier stages when analysing whole body homogenates. So far not enough mechanistic details of the physiological response to increased *p*CO^2^ have been identified to explain the variable findings in different developmental stages of larval fish. Potentially the presence of functional gills^22^ in later developmental stages as well as gut development^37^ for bicarbonate secretion^30^ could be a contributing factor to the observed differences in response between the larval stages.

Similar to the response of a few genes related to the cellular stress response, 36 dph larvae showed some DE genes related to acid-base regulation. Namely Carbonic anhydrase (CA), a zinc metalloenyzme, which catalyses the reaction CO_2_ + H_2_O ↔ H^+^  + HCO_3_^−^ and is crucial for acid-base regulation and CO_2_ excretion in adult fish, was differentially expressed. It is present in a number of isoforms with differences in properties, sequences, and tissue distribution^22,48^. The CA isoform identified to be differentially expressed in this study is CA4. This form is known to be active extracellularly and has a large number of variants in teleost fish^22^. In a previous study, no difference in transcript abundance of any CA4 isoforms was identified in the anterior intestine of adult cod exposed to increased *p*CO_2_^30^. Tseng *et al*.^13^ also identified a downregulation in CA (namely CA2 and CA15) in embryos of medaka under increased *p*CO_2_. But no such differences were identified between hatchlings from a control and a 1200 µatm treatment. Only CA15 transcript abundances at very high *p*CO_2_ (4000 µatm) differed compared to controls. The above study^12^ assessed only selected candidate genes while the expression of CA4 was not addressed. In the present study, CA2 was not found to be DE, while CA15 is not present in the annotation. CA4 may also be involved in stimulating the activity of Na^+^/HCO_3_^−^ co-transporter 1 (NBC1, SLC4A4)^49^, an important mechanism for HCO_3_^−^ transport over the basolateral membrane^23^. In *D. rerio the* expression patterns of the various CA isoforms varies during development from embryo to larvae^41^. As the expression of carbonic anhydrase in cod is likely to vary in a similar pattern it becomes very difficult to disentangle any potential effects of increase *p*CO_2_ and development on CA expression. Interesting from a mechanistic standpoint were also ammonia Rh transporters that are suggested to be important in CO_2_ transport across membranes^50^. All of the identified Rh transporters showed different patterns among larval cod and medaka (Rhcg, Rhcg2, and Rhag)^13^.

For other phenotypic effects such as hepatic steatosis or fatty liver syndrome, an accumulation of lipids in vacuoles in liver cells^14,20^ occurring later during the experiment, we also found some transcriptomic correlates. Insulin induced gene 1 protein (Insig 1), a gene that has been associated with hepatic steatosis in zebrafish^42,51^, showed one of the largest fold changes among the DE genes in the 36 dph larvae (SI Table 6). Individuals exposed to high *p*CO_2_ also showed increased ossification^14^. Accordingly, the upregulated gene col27a1b Collagen alpha-1(XXVII) chain B in 13 dph larvae, associated with calcification of cartilage into bone and particularly within the notochord^52^, (SI Table 3) could potentially be responsible for more ossified vertebrae observed in older larvae^14^.

Is it possible that our study has missed an important transcriptomic response despite high replication (N = 8) and good sequence coverage. To begin with, the use of whole larvae tissue homogenates instead of gill tissues may be suboptimal for detecting expression patterns in ion and acid-base regulation, where we assume that gene expression is strongly tissue specific and could lead to compensatory effects camouflaging difference in gene regulation patterns. However, the skin, solely responsible for acid-base regulation prior to gill formation, is impossible to separate from other tissues for single tissue transcriptome profiling in these early larval stages. Interestingly the number of Na^+^/K^+^ ATPase rich ionocytes (chloride cells) on the yolk sac epithelium of newly hatched cod larvae, important for acid-base base regulation, was not affected by increased *p*CO_2_ either^10^.

More importantly, selection in favour of resilient individuals during the early larval stages may have modulated the patterns of the gene expression analysis across all three examined larval stages, especially in the age groups 6 and 13 dph, were natural and experimental mortality was high. Selection via mortality may have contributed to the low number of genes that were differentially expressed in early larvae, since we are only able to study the survivors. In particular, if damages need to exceed a threshold, genes may have been differentially expressed only for a very short period before larvae die, in which case even the 8-fold replication may have missed a consistent signal.

In conclusion, gene expression patterns in combination with the high mortality^8^ and histological tissue changes^13^ identified together in the same experiment suggest that the predicted end-of-century ocean acidification is not easily detected by early cod larvae as a stressor. This could be related to the fact that functional ion regulatory epithelia in the gill and intestine are not fully developed yet (e.g.^13^). Global RNAseq profiles are supported by target gene data which only show little evidence both qualitatively and quantitatively for the cellular stress response (CSR^34^). Ocean acidification thus seems to constitute a “stealth stressor” that does not trigger classical cellular stress responses but results in tissue damages and increased mortality at a higher level of organismal integration. This is further supported by the lack of genes in the transcriptomic assessment that are indicative of protein damage, cell repair and apoptosis. To fully understand, why no cellular stress response or general transcriptomic response is present, in future experiments moribund larvae should also be sampled in a targeted way and compared to competent larvae to test this hypothesis. More research is needed as to how cellular physiology integrates with tissue growth and development and the physiological integration of the entire organism. Emphasis should also be placed on investigating individual tissues at different developmental stages to assess in higher resolution and pinpoint stages of high vulnerability.

## Methods

### Experimental setup

Adult cod were caught in the Barents Sea (70°15’N, 19°00’E) in March of 2014 and transported to the Centre for Marine Aquaculture, Kvaløya, Norway, where they were transferred into 25 m^2^ spawning tanks equipped with a flow-through of sea water from the fjord of ambient temperature and salinity. Photoperiod was matched to local sunrise and sunset to induce spawning; oxygen saturation, pH, salinity and water temperatures were monitored daily. Once the fish started to spawn, all floating eggs in the spawning tank were collected using a mesh bag behind the surface skimmer. The volume of collected eggs was divided into two equal portions and moved into 28 L flow-through incubators. Half of the egg incubators were adjusted to ambient (503 ± 89 μatm) and the other half to end-of-century (1179 ± 87 μatm) *p*CO_2_ concentration.

Two days after >50% of larvae had hatched (same day in both treatments), approximately 11 000 larvae were transferred into each of six 190 L rearing tanks, three replicates of each *p*CO_2_ treatment. All larvae were fed using a reduced aquaculture feeding regime for commercial fry production (termed low food treatment in^9,14^), starting with green water addition (*Nannochloropsis*) followed by enriched *Brachionus* and later *Artemia* nauplii (See “Low food” in^14^ Table 1). The water temperature for the rearing tanks was initially kept at 6 °C but was increased to 10 °C after day 6, while the photoperiod was kept constant throughout the experiment (24 h light). For larval weight analysis, 12 larvae from each replicate were sampled at day 5, 15 and 36 days-post-hatch (dph), euthanized using MS-222 (Tricaine methanesulfonate, 0.04% solution (m/V)) and frozen in treatment water at −20 °C. Single larvae for transcriptome analysis were sampled randomly from all tanks at 6, 13 and 36 dph, immediately euthanized using MS-222 and submersed in RNA-later®, placed at 8 °C for 24 h and subsequently stored in –78 °C until RNA isolation.

We continually monitored CO_2_ concentrations in both treatments using pH sensors as proxy to identified potential changes of *p*CO_2_. Data were automatically stored on a computer system (Aquastar, IKS Computer systems, Karlsbad, Germany). Increased *p*CO_2_ concentrations in the water for the end-of-century treatment were achieved by aerating the header tanks with CO_2_, which ensured equal and constant *p*CO_2_ concentrations in all replicates of both ambient and high *p*CO_2_ treatments. The absorption of CO_2_ into the header tanks was regulated by a magnetic valve that controlled the aeration with CO_2_ by the IKS system through pH-sensors in the outflow of the header tank. Further, temperature, salinity and pH were measured daily with a hand held multi probe (WTW pH/Cond 340i/3320). Additionally, the *p*CO_2_ levels in the tanks and incubators were checked weekly from calculated *p*CO_2_ using total dissolved inorganic carbon and total alkalinity and the chemical speciation calculation program CO2SYS^53^, in accordance to the Best practice guide^54^. The mean *p*CO_2_ values and standard deviation for the ambient and the high *p*CO_2_ treatment were 503 ± 89 and 1179 ± 87 μatm, respectively, corresponding to an *in situ* pH of 8.00 and 7.68, respectively. For more detailed information please consult the Supplementary Material of^9^.

### Ethics statement

This study was carried out at NOFIMA’s Centre for Marine Aquaculture, Kvaløya, Norway applying methods and protocols approved by the National Regulatory Committee on the Ethics of Animal Experiments, Norway under the permit number: FOTS id 6382 and in strict accordance to the relevant regulations and guidelines. All possible actions were taken to reduce animals suffering and stress during handling and sampling.

### Larval dry weight

Vials containing larvae for dry weight analysis were thawed on ice; the individual larvae were inspected for completeness under a stereomicroscope, rinsed in distilled water and placed in individual vials. Larvae were freeze dried for 16 h before being weighed to the closest 0.1 µg. Damaged and incomplete larvae were excluded from the analysis. The statistical analysis was performed in R version 3.3.2^55^, using R studio^56^ and a linear mixed model (R-package lme4^57^). Larval treatment as a fixed factor and tank as a random factor was applied for each of the 5 and 15-day old age groups. The data for 36 dph were analysed and presented in reference^14^. These sampling days were chosen for their proximity to the RNA-seq sampling points.

### RNA isolation and sample preparation

We chose to investigate the global transcriptome of homogenates of whole, single cod larvae at several time points, instead of the often-used gill tissue, as organ development is constantly changing in these life stages and gills are not developed at hatch. The rational for studying the 3 different age groups was based on (1) using first feeding larvae (5–6 dph), which had successfully made the change from internal to external feeding and were the articulation of the skeletal structures of the jaw had occurred^36,37^. (2) established feeding larvae at an age (13–15 dph) were high mortality in relation to OA treatment had been observed ^9^and (3) larvae (36 dph), which had made the transition from cutaneous to branchial respiration, showing large numbers of gill filament, existence of a gill cover and bone ossification having started^14,37^.

Larvae preserved in RNALater® were thawed on ice, dapped dry on paper tissue and wet weight measured to the closest 0.001 mg. RNA from individual larvae was isolated using the RNeasy Kit (Qiagen, Hilden, Germany) with a modified protocol implementing on-column DNase digestion steps (RNase-Free DNase Set, Qiagen, Hilden, Germany). Briefly, the whole larva was placed in the lysis solution immediately after thawing and homogenized using a TissueLyserII and glass beads (Qiagen, Hilden, Germany) for 2 min at 20 Hz before 600 µl 70% EtOH was added. 700 µl of the homogenate were transferred to the spin column and centrifuged at 8000 g for 15 sec. The flow-through was discarded (as in all following steps except elution) and the remaining 500 µl of the homogenate was added to the column before the centrifugation process was repeated. A wash step was performed before the on-column DNA digestion for 15 min at room temperature. Three additional washing steps were performed as per manufacturer specification before the column was centrifuged for 2 min at maximal speed to remove all remains of the wash buffers and to be dried. The RNA was eluted from the column using 50 µl of RNase and DNase free water for 1 min at 8000 × g. Purity of the RNA extract was assessed by spectrophotometry (ND-1000, ThermoFisher Scientific, Waltham, MA, United States), quantified with a broad range RNA test on a fluorimeter (Qubit2, ThermoFisher Scientific, Waltham, MA, United States) and the RNA integrity was evaluated with a Eukaryote Total RNA StdSens chip using an Experion automated electrophoresis system (Bio-Rad, Hercules, CA, United States). Samples with purity, quantity and integrity below the recommendations and guidelines of the sequencing centre were excluded from further analysis.

### Sequencing

In total, 48 mRNA (8 biological replicates per treatment (ambient and high *p*CO_2_) and 3 sampling points (6, 13 and 36 dph) cDNA libraries were constructed using the TruSeq Stranded mRNAseq Sample Prep kit (Illumina Inc., San Diego, CA, United States; 0.5 µg total RNA input). Sequencing was performed on a HiSeq4000 (Illumina Inc., San Diego, CA, United States) platform with a 150 bp paired-end protocol. After quality control of the library preparations, all samples were pooled and run across 5 lanes.

After an initial round of quality control, we detected too low sequencing depth (around 20% of average) in 5 samples, which were then re-sequenced on an additional lane to supplement the previously sequenced data to satisfactory levels.

### Bioinformatics and statistical data analysis

The sequencing adaptors were removed using Trimmomatic Version 0.36^58^. Of the resulting data, all unpaired reads were discarded and only paired reads were used for further analysis. The quality of the remaining reads was assessed with FastQC^59^ and MultiQC^60^. All paired reads were compared to a transcriptome created, using getfasta of bedtools^61^ based on the most recent published cod genome and its annotations^62^, using Kallisto^63^. Transcript abundance data generated by Kallisto were imported into R studio^55,56^ using the tximport package^64^. The differential gene expression (DE) analysis was performed using DESeq2^65^ using default settings with negative binomial distribution data normalization and a FDR (false recovery rate) correction using the Benjamini-Hochberg (BH) method^66^.

A gene ontology (GO) enrichment analysis was performed using GOseq^67^ for all genes that were found to be significantly up or down regulated in the DE analysis at 36 dph. Further, candidate genes were extracted from the existing literature, focusing on genes related to acid-base regulation^23,29^ and genes related to the minimal stress response^35^. All data visualizations were created using the ggplot2^68^ or DESeq2^65^ packages in R^55^.

Read counts assigned to the reference were transformed using the regularized logarithm (rlog)^65^ to be used in multivariate analysis, including principal component analysis (PCA) using prcomp, to visualize clustering of samples and to assess the underlying loadings. Further an analysis of similarity (ANOSIM) in R-package vegan^69^ was performed on Euclidian distances to assess differences between the age groups.

## Supplementary information

Supplementary information

## Data Availability

RNA-Seq data has been deposited to the NCBI Gene expression omnibus (GEO) under https://www.ncbi.nlm.nih.gov/geo/query/acc.cgi?acc=GSE108715, growth data (ages 5 and 15 dph) has been submitted to Pangea 10.1594/PANGAEA.884548. DIC data is available under 10.1594/PANGAEA.858615.
