# Supplementary material for: Transcriptome profiling reveals exposure to predicted end-of-century ocean acidification as a stealth stressor for Atlantic cod larvae
Source: Sci Rep. 2019 Nov 15;9:16908. doi: 10.1038/s41598-019-52628-1 (PMC6858462; doi:10.1038/s41598-019-52628-1)
Supplement: Supplementary file 1 — Supplementary information [file 41598_2019_52628_MOESM1_ESM.pdf]

Supplementary information

Transcriptome profiling reveals exposure to predicted end-of-century ocean acidification as a stealth stressor for Atlantic cod larvae

F.H. Mittermayer[1*], M.H. Stiasny[1,2], C. Clemmesen[1], T. Bayer[1], V. Puvanendran[3], M. Chierici[4], S. Jentoft[5] & T.B.H. Reusch[1]

[1]GEOMAR Helmholtz Centre for Ocean Research Kiel, Marine Evolutionary Ecology, Düsternbrooker Weg 20, 24105 Kiel, Germany

[2]Dept. of Economics, Kiel University, Sustainable Fisheries, Wilhelm-Seelig-Platz 1, 24118 Kiel, Germany

[3]Nofima AS, Muninbakken 9, NO-9019 Tromsø, Norway

[4]Institute for Marine Research, Framsenteret, Hjalmar Johansens gate 14, NO-9007 Tromsø, Norway

[5]Centre for Ecological and Evolutionary Synthesis (CEES), Department of Biosciences, University of Oslo, Postboks 1066, NO-0316 Oslo, Norway

*Corresponding author: fmittermayer@geomar.de

Table 1: Results of a linear mixed model for dry weight of 5 and 15 dph cod larvae, including tank as a random factor

Larval age	Response	Fixed factor	F	DF(num)	DF(denum)	p-value
5 dph	Dry weight	Larval pCO_2 (ambient/increased)	6.0045	1	3.725	0.075
15 dph	Dry weight	Larval pCO_2 (ambient/increased)	2.0643	1	3	0.246

Table 2. Summary of samples, treatments, sequencing and assignment results. # presents number, SD is standard deviation. Read values refer to single reads, raw reads are total number of reads per sampling category, and filtered reads are average per sample.

			RAW READS DE-MULTIPLEXED			FILTERED READS USED IN DEANALYSIS		ASSIGNED UNIQUELY TO THE REF. TRANSCRIPTOME	
**Treatment**	**Age (dph)**	**# Samples**	**Sum**	**Mean #**	**SD**	**Mean #**	**SD**	**Mean %**	**SD**
**Ambient pCO_2**	**6**	**8**	**$5.1*10^8$**	**$6.4*10^7$**	**$1.8*10^5$**	**$6.4*10^7$**	**$1.8*10^5$**	**70.58**	**1.00**
**High pCO_2**	**6**	**8**	**$5.3*10^8$**	**$6.6*10^7$**	**$2.8*10^5$**	**$6.5*10^7$**	**$2.8*10^5$**	**70.52**	**0.96**
**Ambient pCO_2**	**13**	**8**	**$6.2*10^8$**	**$7.8*10^7$**	**$24.3*10^5$**	**$7.7*10^7$**	**$24.2*10^5$**	**71.11**	**0.75**
**High pCO_2**	**13**	**8**	**$5.2*10^8$**	**$6.5*10^7$**	**$4.8*10^5$**	**$6.4*10^7$**	**$4.7*10^5$**	**71.29**	**1.35**
**Ambient pCO_2**	**36**	**8**	**$6.9*10^8$**	**$8.6*10^7$**	**$28.5*10^5$**	**$8.4*10^7$**	**$28.2*10^5$**	**61.23**	**2.88**
**High pCO_2**	**36**	**8**	**$5.4*10^8$**	**$6.7*10^7$**	**$2.8*10^5$**	**$6.7*10^7$**	**$2.7*10^5$**	**70.51**	**2.92**
**Total**		**48**	**$34.1*10^8$**	**$7.1*10^7$**		**$7.0*10^7$**		**69.20**	

Table 3a. Loadings of first principal component, 25 main contributing genes to first principle component in Figure 3

**GENE**	**CONTRIBUTION TO PC 1 IN %**
**MYOSIN HEAVY CHAIN. FAST SKELETAL MUSCLE (*CYPRINUS CARPIO*)**	0.388066021
**KLHL38 KELCH-LIKE PROTEIN 38 (*DANIO RERIO*)**	0.371193025
**FBXO32 F-BOX ONLY PROTEIN 32 (*SUS SCROFA*)**	0.302487707
**MYOSIN HEAVY CHAIN. FAST SKELETAL MUSCLE (*CYPRINUS CARPIO*)**	0.301755979
**PROTEIN OF UNKNOWN FUNCTION**	0.237395845
**GAMMA-CRYSTALLIN M3 (*CYPRINUS CARPIO*)**	0.223435332
**PROTEIN OF UNKNOWN FUNCTION**	0.218408605
**LADDERLECTIN (*ONCORHYNCHUS MYKISS*)**	0.217564849
**FAM134B RETICULOPHAGY RECEPTOR FAM134B (*MUS MUSCULUS*)**	0.198892372
**PROTEIN OF UNKNOWN FUNCTION**	0.182636968
**PROTEIN OF UNKNOWN FUNCTION**	0.181408566
**MYOSIN HEAVY CHAIN. FAST SKELETAL MUSCLE (*CYPRINUS CARPIO*)**	0.175043319
**ARRDC2 ARRESTIN DOMAIN-CONTAINING PROTEIN 2 (*HOMO SAPIENS*)**	0.170943549
**IGFBP1 INSULIN-LIKE GROWTH FACTOR-BINDING PROTEIN 1 (*BOS TAURUS*)**	0.170560436
**VCAN VERSICAN CORE PROTEIN (*GALLUS GALLUS*)**	0.168464054
**EXOSC6 EXOSOME COMPLEX COMPONENT MTR3 (*DANIO RERIO*)**	0.162277631
**UCP2 MITOCHONDRIAL UNCOUPLING PROTEIN 2 (*MUS MUSCULUS*)**	0.161029997
**VISININ (*GALLUS GALLUS*)**	0.160272963
**BNIP3 BCL2/ADENOVIRUS E1B 19 KDA PROTEIN-INTERACTING PROTEIN 3 (*MUS MUSCULUS*)**	0.159401846
**VERRUCOTOXIN SUBUNIT BETA (*SYNANCEIA VERRUCOSA*)**	0.154615965
**C4 COMPLEMENT C4 (*RATTUS NORVEGICUS*)**	0.15237641
**HMGCS1 HYDROXYMETHYLGLUTARYL-COA SYNTHASE. CYTOPLASMIC (*GALLUS GALLUS*)**	0.1502471
**COL9A3 COLLAGEN ALPHA-3(IX) CHAIN (*GALLUS GALLUS*)**	0.149620902
**CYP51A1 LANOSTEROL 14-ALPHA DEMETHYLASE (*MACACA FASCICULARIS*)**	0.145205011

Table 3b. Loadings of first principal component, 25 main contributing genes to second principle component in Figure 3

**GENE**	**CONTRIBUTION TO PC 2 IN %**
**ES1 ES1 PROTEIN. MITOCHONDRIAL (*DANIO RERIO*)**	0.631738076
**MYOSIN HEAVY CHAIN. FAST SKELETAL MUSCLE (*CYPRINUS CARPIO*)**	0.518263622
**MRC1 MACROPHAGE MANNOSE RECEPTOR 1 (*HOMO SAPIENS*)**	0.357842258
**HCEA HIGH CHORIOLYTIC ENZYME 1 (*ORYZIAS LATIPES*)**	0.315547116
**PROTEIN OF UNKNOWN FUNCTION**	0.292950430
**IMPA1 INOSITOL MONOPHOSPHATASE 1 (*BOS TAURUS*)**	0.284643925
**C3 COMPLEMENT C3 (FRAGMENT) (*ONCORHYNCHUS MYKISS*)**	0.282401366
**COL10A1 COLLAGEN ALPHA-1(X) CHAIN (*GALLUS GALLUS*)**	0.246564047
**EXOSC6 EXOSOME COMPLEX COMPONENT MTR3 (*DANIO RERIO*)**	0.228810163
**PROTEIN OF UNKNOWN FUNCTION**	0.201614736
**PROTEIN OF UNKNOWN FUNCTION**	0.186421801
**PEG3 PATERNALLY-EXPRESSED GENE 3 PROTEIN (*BOS TAURUS*)**	0.179113111
**ELASTASE-1 (*SALMO SALAR*)**	0.179070813
**RPL31 60S RIBOSOMAL PROTEIN L31 (*PARALICHTHYS OLIVACEUS*)**	0.177788248
**ENTPD5 ECTONUCLEOSIDE TRIPHOSPHATE DIPHOSPHOHYDROLASE 5 (*GALLUS GALLUS*)**	0.177782763
**COL1A2 COLLAGEN ALPHA-2(I) CHAIN (*RATTUS NORVEGICUS*)**	0.176887258
**VCAN VERSICAN CORE PROTEIN (*GALLUS GALLUS*)**	0.174817539
**CKMT1 CREATINE KINASE U-TYPE. MITOCHONDRIAL (*GALLUS GALLUS*)**	0.173735764
**LECT2 LEUKOCYTE CELL-DERIVED CHEMOTAXIN-2 (*HOMO SAPIENS*)**	0.169742346
**CELA3B CHYMOTRYPSIN-LIKE ELASTASE FAMILY MEMBER 3B (*MUS MUSCULUS*)**	0.164459664
**INTERMEDIATE FILAMENT PROTEIN ON3 (*CARASSIUS AURATUS*)**	0.164073293
**PROTEIN OF UNKNOWN FUNCTION**	0.163405256
**C3 COMPLEMENT C3 (FRAGMENT) (*ONCORHYNCHUS MYKISS*)**	0.158777821
**ITIH3 INTER-ALPHA-TRYPSIN INHIBITOR HEAVY CHAIN H3 (*MUS MUSCULUS*)**	0.156855282

Table 4: List of all differentially expressed genes in 6 dph larvae between ambient (~503µatm) and high (~1179 µatm) pCO_2

Gene (gene name in uniprot/swissprot)	Involved in	log_2 Fold Change	L_2 FC SE	p-value	p adjusted (FDR=BH)
**MEP1B Meprin A subunit beta (Homo sapiens)**	Hydrolase, Metalloprotease, Protease, Inflammatory response	-0.38	0.08	1.45E-06	0.01373
**Ugt2a2 UDP-glucuronosyltransferase 2A2 (Mus musculus)**	Glycosyltransferase, Transferase	-0.34	0.07	8.66E-07	0.01373
**ANKRD6 Ankyrin repeat domain-containing protein 6 (Homo sapiens)**	Positive JNK cascade regulation, Wnt Pathway	-0.39	0.08	1.81E-06	0.01373

Table 5: List of all differentially expressed genes in 13 dph larvae between ambient (~503µatm) and high (~1179 µatm)

Gene (gene name in uniprot/swissprot)	Involved in	log_2 Fold Change	L_2 FC SE	p-value	p adjusted (FDR=BH)
**ANXA5 Annexin A5 (Macaca fascicularis)**	Blood coagulation, Calcium ion binding	0.91	0.11	3.11E-17	7.10E-13
**Protein of unknown function**		0.54	0.11	3.87E-07	0.00441
**blm Bloom syndrome protein homolog (Xenopus laevis)**	DNA-binding, DNA replication	-0.48	0.10	8.18E-07	0.00622
**zpld1 Zona pellucida-like domain-containing protein 1 (X. laevis)**	Membrane component	0.49	0.10	3.45E-06	0.01310
**C1QL4 Complement C1q-like protein 4 (Homo sapiens)**	Identical protein binding, negative regulation fat cell differentiation and fibroblast proliferation	0.51	0.11	2.95E-06	0.01310
**CILP2 Cartilage intermediate layer protein 2 (Homo sapiens)**	Extra cellular matrix	0.45	0.10	2.82E-06	0.01310
**Nol6 Nucleolar protein 6 (Mus musculus)**	RNA-binding, RNA transport	-0.51	0.11	4.40E-06	0.01434
**KIAA1586 Uncharacterized protein KIAA1586 (Homo sapiens)**	Ligase activity	-0.48	0.11	1.25E-05	0.03057
**col27a1b Collagen alpha-1(XXVII) chain B (Danio rerio)**	Calcification of cartilage, bone development	0.42	0.10	1.60E-05	0.03057

**GATM Glycine amidinotransferase, mitochondrial (Gallus gallus)**	amidinotransferase activity	-0.42	0.10	1.69E-05	0.03057
**NR4A3 Nuclear receptor subfamily 4 group A member 3 (Homo sapiens)**	DNA binding, Transcript regulation	-0.48	0.11	1.45E-05	0.03057
**Irg1 Cis-aconitate decarboxylase (Mus musculus)**	Antimicrobial, Inflammatory response	-0.43	0.10	1.88E-05	0.03057
**Protein of unknown function**		-0.49	0.11	1.24E-05	0.03057
**comA Comitin (Dictyostelium discoideum)**	Actin-binding	-0.46	0.11	1.81E-05	0.03057
**SPR Sepiapterin reductase (Homo sapiens)**	Oxidoreductase	-0.39	0.09	2.69E-05	0.04090
**Trypsin I-P1 (Gallus gallus)**	Hydrolase, Protease, Digestion	0.46	0.11	2.97E-05	0.04235

Table 6: List of all differentially expressed genes with L2FC ≤-1/≥1 in 36 dph larvae between ambient (~503µatm) and high (~1179 µatm)

Gene (gene name in uniprot/swissprot)		log_2 Fold Change	L_2 FC SE	p-value	p adjusted (FDR=BH)
**Fosb Protein fosB (Mus musculus)**	DNA binding	-2.29	0.18	1.22E-37	2.35E-33
**Klf4 Krueppel-like factor 4 (M. musculus)**	DNA binding, Transcription regulation	-1.79	0.18	1.26E-23	8.13E-20
**ATF3 Cyclic AMP-dependent transcription factor ATF-3 (Homo sapiens)**	DNA binding, Transcription regulation	-1.68	0.17	2.05E-23	9.87E-20
**Ptchd3 Patched domain-containing protein 3 (M. musculus)**	Membrane component	-1.54	0.22	6.74E-12	9.28E-09
**lin28a Protein lin-28 homolog A (Danio rerio)**	RNA binding, gene silencing	-1.33	0.20	1.49E-11	1.92E-08
**AREG Amphiregulin (Homo sapiens)**	Growth factor	-1.33	0.15	2.37E-19	7.63E-16
**KLF2 Krueppel-like factor 2 (Homo sapiens)**	DNA binding, Transcription regulation	-1.30	0.11	2.98E-34	2.87E-30
**phlda2 Pleckstrin homology-like domain family A member 2 (Salmo salar)**	Membrane component	-1.18	0.12	2.55E-22	9.84E-19
**Fosl1 Fos-related antigen 1 (Rattus norvegicus)**	DNA binding	-1.18	0.21	4.24E-08	2.21E-05
**Trim39 E3 ubiquitin-protein ligase TRIM39 (M.s musculus)**	Identical protein binding, Transferase	-1.17	0.22	6.95E-08	3.11E-05
**Protein of unknown function**		-1.13	0.17	3.55E-11	4.03E-08
**PTGER4 Prostaglandin E2 receptor EP4 subtype (Homo sapiens)**	Numerous functions	-1.08	0.22	1.46E-06	0.00032
**Sgms2 Phosphatidylcholine:ceramide cholinephosphotransferase 2 (R. norvegicus)**	Membrane component	-1.03	0.15	4.30E-12	6.92E-09

**Myosin heavy chain. fast skeletal muscle (Cyprinus carpio)**	Actin binding muscle protein	1.00	0.22	6.23E-06	0.00101
**Protein of unknown function**		1.01	0.22	6.53E-06	0.00103
**ADPRH [Protein ADP-ribosylarginine] hydrolase (Homo sapiens)**	Cellular protein modification	1.01	0.21	1.53E-06	0.00033
**insig1 Insulin-induced gene 1 protein (Xenopus tropicalis)**	Lipid and Cholesterol metabolism	1.04	0.20	3.68E-07	0.00011
**ENTPD5 Ectonucleoside triphosphate diphosphohydrolase 5 (Gallus gallus)**	Cell growth and proliferation	1.06	0.22	1.01E-06	0.00025
**Protein of unknown function**		1.06	0.22	2.29E-06	0.00045

Table 7: List of differentially expressed candidate genes related to acid-base regulation and stress response in 36 dph larvae between ambient (~503 µatm) and high pCO_2 treatment (~1179 µatm).

Name	Group	Source reference	Annotation name (uniprot/swissprot)	log2 FoldChange	p value	padj
A3 SLC family 26 (SLC26A3)	Acid-Base	Micheal et al., 2015	Slc26a3 Chloride anion exchanger (*Rattus norvegicus)*	-0,3846619	0,02075377	0,13462717
A3 SLC family 26 (SLC26A3)	Acid-Base	Micheal et al., 2015	Slc26a3 Chloride anion exchanger (*Rattus norvegicus)*	0,0600537	0,73268794	0,87740277
A6 SLC family 26 (SLC26A6)	Acid-Base	Micheal et al., 2015	Slc26a6 Solute carrier family 26 member 6 (*Mus musculus*)	-0,1109202	0,41296842	0,67135219
A6 SLC family 26 (SLC26A6)	Acid-Base	Micheal et al., 2015	Slc26a6 Solute carrier family 26 member 6 (*Mus musculus*)	-0,4247676	0,00640451	0,06972912
A6 SLC family 26 (SLC26A6)	Acid-Base	Micheal et al., 2015	Slc26a6 Solute carrier family 26 member 6 (*Mus musculus*)	-0,1963062	0,22446881	0,49480152

AE1 SLC4a1 (HCO3 transporter)	Acid-Base	Heuer&Grossell, 2014	slc4a1 Band 3 anion exchange protein (*Oncorhynchus mykiss*)	0,08014627	0,60939462	0,81080286	
AE2 SLC4a2 (HCO3 transporter)	Acid-Base	Heuer&Grossell, 2014	SLC4A2 Anion exchange protein 2 (*Homo sapiens)*	0,11318606	0,15677774	0,40898133	
AE2 SLC4a2 (HCO3 transporter)	Acid-Base	Heuer&Grossell, 2014	SLC4A2 Anion exchange protein 2 (*Homo sapiens*)	-0,1728193	0,04767664	0,21370794	
CA (carbonic anhydras)	Acid-Base	Heuer&Grossell, 2014	ca1 Carbonic anhydrase 1 (*Chionodraco hamatus*)	0,26481502	0,05228608	0,22502006	
CA (carbonic anhydras)	Acid-Base	Heuer&Grossell, 2014	CA14 Carbonic anhydrase 14 (*Homo sapiens*)	0,00280535	0,97561354	0,99014811	
CA (carbonic anhydras)	Acid-Base	Heuer&Grossell, 2014	CA4 Carbonic anhydrase 4 (*Homo sapiens*)	-0,2938201	0,04929332	0,21780218	
CA (carbonic anhydras)	Acid-Base	Heuer&Grossell, 2014	CA4 Carbonic anhydrase 4 (*Homo sapiens*)	0,24796459	0,12511754	0,36278252	
CA (carbonic anhydras)	Acid-Base	Heuer&Grossell, 2014	Ca4 Carbonic anhydrase 4 (*Rattus norvegicus*)	-0,5427883	1,6746E-05	0,00192171	*
CA (carbonic anhydras)	Acid-Base	Heuer&Grossell, 2014	CA5B Carbonic anhydrase 5B, mitochondrial (*Homo sapiens*)	-0,0567403	0,75825013	0,89093809	
CA (carbonic anhydras)	Acid-Base	Heuer&Grossell, 2014	CA6 Carbonic anhydrase 6 (*Homo sapiens*)	0,11641642	0,1739237	0,43248743	
CA (carbonic anhydras)	Acid-Base	Heuer&Grossell, 2014	CA7 Carbonic anhydrase 7 (*Homo sapiens*)	-0,2156911	0,22043977	0,4897843	
CA (carbonic anhydras)	Acid-Base	Heuer&Grossell, 2014	cahz Carbonic anhydrase (*Danio rerio*)	-0,0867529	0,57359484	0,78705972	
CA (carbonic anhydras)	Acid-Base	Heuer&Grossell, 2014	CA12 Carbonic anhydrase 12 (*Homo sapiens*)	-0,0285542	0,89257103	NA	
NBC1 (Na/HCO3 contransporter 1) SLC4A4	Acid-Base	Heuer&Grossell, 2014	Slc4a4 Electrogenic sodium bicarbonate cotransporter 1 (*Mus musculus*)	-0,0477387	0,81337672	0,91725253	
NBC1 (Na/HCO3 contransporter 1) SLC4A4	Acid-Base	Heuer&Grossell, 2014	SLC4A4 Electrogenic sodium bicarbonate cotransporter 1 (*Oryctolagus cuniculus*)	-0,0728577	0,1597365	0,41313037	
NHE1 (Na/H exchanger)SLC9A1	Acid-Base	Heuer&Grossell, 2014	SLC9A1 Sodium/hydrogen exchanger 1 (*Oryctolagus cuniculus*)	-0,0757678	0,64436079	0,83123418	

NHE2 (Na/H exchanger) SLC9 member 2	Acid-Base	Heuer&Grossell, 2014	SLC9A2 Sodium/hydrogen exchanger 2 (*Oryctolagus cuniculus*)	0,0485859	0,70673443	0,86437437
NHE2 (Na/H exchanger) SLC9 member 2	Acid-Base	Heuer&Grossell, 2014	Slc9a2 Sodium/hydrogen exchanger 2 (*Rattus norvegicus*)	-0,0892483	0,66849604	NA
NHE3 (Na/H exchanger) SLC9A3	Acid-Base	Heuer&Grossell, 2014	SLC9A3 Sodium/hydrogen exchanger 3 (*Didelphis virginiana*)	-0,0820974	0,68420678	0,85405131
NHE3 (Na/H exchanger) SLC9A3	Acid-Base	Heuer&Grossell, 2014	Slc9a3 Sodium/hydrogen exchanger 3 (*Rattus norvegicus*)	-0,1747312	0,23027821	0,50164221
NKA Na/K ATPase	Acid-Base	Heuer&Grossell, 2014	ATP1A1 Sodium/potassium-transporting ATPase subunit alpha-1 (*Bos taurus*)	0,15435617	0,04064224	0,19608152
NKA Na/K ATPase	Acid-Base	Heuer&Grossell, 2014	ATP1A1 Sodium/potassium-transporting ATPase subunit alpha-1 (*Bos taurus*)	-0,0531942	0,69609749	0,86064667
NKA Na/K ATPase	Acid-Base	Heuer&Grossell, 2014	ATP1A1 Sodium/potassium-transporting ATPase subunit alpha-1 (*Pongo abelii*)	-0,0438916	0,64592473	0,83222417
NKA Na/K ATPase	Acid-Base	Heuer&Grossell, 2014	ATP1A2 Sodium/potassium-transporting ATPase subunit alpha-2 (*Sus scrofa*)	-0,108302	0,52712569	0,75748779
NKA Na/K ATPase	Acid-Base	Heuer&Grossell, 2014	atp1a3 Sodium/potassium-transporting ATPase subunit alpha-3 (*Oreochromis mossambicus*)	0,23089579	0,00888909	0,08414143
NKA Na/K ATPase	Acid-Base	Heuer&Grossell, 2014	ATP1B2 Sodium/potassium-transporting ATPase subunit beta-2 (*Homo sapiens*)	0,14173092	0,17917111	0,43954238
NKA Na/K ATPase	Acid-Base	Heuer&Grossell, 2014	ATP1B2 Sodium/potassium-transporting ATPase subunit beta-2 (*Homo sapiens*)	0,02628338	0,84745887	0,93270306
NKA Na/K ATPase	Acid-Base	Heuer&Grossell, 2014	atp1b3 Sodium/potassium-transporting ATPase subunit beta-3 (*Xenopus laevis*)	0,05616159	0,50566192	0,74405862
NKCC1 (Na/K/Cl cotransporter)SLC12A2	Acid-Base	Heuer&Grossell, 2014	SLC12A2 Solute carrier family 12 member 2 (*Homo sapiens*)	0,03271988	0,63205002	0,8247795
NKCC1 (Na/K/Cl cotransporter)SLC12A2	Acid-Base	Heuer&Grossell, 2014	Slc12a2 Solute carrier family 12 member 2 (*Mus*	0,1074674	0,48685514	0,72845016

			musculus)				
NKCC1 (Na/K/Cl cotransporter)SLC12A2	Acid-Base	Heuer&Grossell, 2014	Slc12a2 Solute carrier family 12 member 2 (*Mus musculus*)	0,11312732	0,33450782	0,60434602	
RH transporter	Acid-Base-related	Heuer&Grossell, 2014	Similar to RHAG Ammonium transporter Rh type A (*Homo sapiens*)	0,14296829	0,05392902	0,2284024	
RH transporter	Acid-Base-related	Heuer&Grossell, 2014	Similar to Rhag Ammonium transporter Rh type A (*Mus musculus*)	-0,5249972	0,00758553	0,0765662	
RH transporter	Acid-Base-related	Heuer&Grossell, 2014	Similar to rhbg Ammonium transporter Rh type B (*Takifugu rubripes*)	0,4042237	0,00174209	0,03315483	*
RH transporter	Acid-Base-related	Heuer&Grossell, 2014	Similar to rhbg Ammonium transporter Rh type B (*Xenopus tropicalis*)	0,04694015	0,82553266	0,92258096	
RH transporter	Acid-Base-related	Heuer&Grossell, 2014	Similar to rhbg-a Ammonium transporter Rh type B-A (*Xenopus laevis*)	0,06444727	0,43681513	0,68998518	
RH transporter	Acid-Base-related	Heuer&Grossell, 2014	Similar to rhcg Ammonium transporter Rh type C (*Tetraodon nigroviridis*)	-0,128581	0,52099571	0,75394883	
RH transporter	Acid-Base-related	Heuer&Grossell, 2014	Similar to rhcg2 Ammonium transporter Rh type C 2 (*Takifugu rubripes*)	-0,3687006	0,00047045	0,01599065	*
6 phosphogluconate dehydrogenase	Stress	Kültz, 2005	PGD 6-phosphogluconate dehydrogenase, decarboxylating (*Homo sapiens*)	0,09998854	0,58764526	0,79727045	
Aldehyde dehydrogenase	Stress	Kültz, 2005	ALDH16A1 Aldehyde dehydrogenase family 16 member A1 (*Bos taurus*)	0,04050142	0,61711186	0,81583347	
Aldehyde dehydrogenase	Stress	Kültz, 2005	ALDH1A3 Aldehyde dehydrogenase family 1 member A3 (*Homo sapiens*)	0,390955	0,00111631	0,0262097	*
Aldehyde dehydrogenase	Stress	Kültz, 2005	ALDH2 Aldehyde dehydrogenase, mitochondrial (*Mesocricetus auratus*)	0,09585625	0,41507678	0,67302483	
Aldehyde dehydrogenase	Stress	Kültz, 2005	ALDH3A2 Fatty aldehyde dehydrogenase (*Macaca fascicularis*)	-0,0484776	0,7329881	0,87744661	

Aldehyde dehydrogenase	Stress	Kültz, 2005	ALDH3A2 Fatty aldehyde dehydrogenase (*Macaca fascicularis*)	0,0053013	0,94620884	0,97812119	
Aldehyde dehydrogenase	Stress	Kültz, 2005	Aldh6a1 Methylmalonate-semialdehyde dehydrogenase [acylating], mitochondrial (*Mus musculus*)	0,15815337	0,2964247	0,5679305	
Aldehyde dehydrogenase	Stress	Kültz, 2005	Aldh6a1 Methylmalonate-semialdehyde dehydrogenase [acylating], mitochondrial (*Mus musculus*)	0,00319833	0,98000719	0,99178785	
Aldehyde dehydrogenase	Stress	Kültz, 2005	Aldh6a1 Methylmalonate-semialdehyde dehydrogenase [acylating], mitochondrial (*Rattus norvegicus*)	0,21835788	0,02559703	0,15171187	
Aldehyde dehydrogenase	Stress	Kültz, 2005	ALDH7A1 Alpha-aminoadipic semialdehyde dehydrogenase (*Bos taurus*)	0,13771501	0,35701142	0,62392812	
Aldehyde dehydrogenase	Stress	Kültz, 2005	aldh8a1 Aldehyde dehydrogenase family 8 member A1 (*Danio rerio*)	-0,1634678	0,33560169	0,60575461	
Aldehyde dehydrogenase	Stress	Kültz, 2005	aldh9A1 Betaine aldehyde dehydrogenase (*Gadus morhua subsp. callarias*)	0,2732407	0,10537731	0,33055144	
Aldehyde dehydrogenase	Stress	Kültz, 2005	aldh9a1b Aldehyde dehydrogenase family 9 member A1-B (*Danio rerio*)	0,16217874	0,14447711	0,3923615	
Aldehyde reductase	Stress	Kültz, 2005	AKR1B10 Aldo-keto reductase family 1 member B10 (*Homo sapiens*)	0,22983971	0,00351227	0,04882458	*
Aldehyde reductase	Stress	Kültz, 2005	Akr7a2 Aflatoxin B1 aldehyde reductase member 2 (*Rattus norvegicus*)	0,05875718	0,64148548	0,82945664	
Aminobutyrate aminotransferase	Stress	Kültz, 2005	ABAT 4-aminobutyrate aminotransferase, mitochondrial (*Homo sapiens*)	-0,1419473	0,06935656	0,26368077	
Aromatic amino acid aminotransferase	Stress	Kültz, 2005	very diverse, not included in analysis	no data	no data	no data	
Ca2+/Mg2+-transporting ATPase	Stress	Kültz, 2005	ATP2C1 Calcium-transporting ATPase type 2C member 1 (*Homo sapiens*)	-0,1908614	0,15400883	0,40555311	

Citrate synthase (Krebs cycle)	Stress	Kültz, 2005	ACLY ATP-citrate synthase (*Homo sapiens*)	0,17301081	0,02634519	0,15349318
Citrate synthase (Krebs cycle)	Stress	Kültz, 2005	Acly ATP-citrate synthase (*Mus musculus*)	0,15680517	0,23542998	0,50730124
Citrate synthase (Krebs cycle)	Stress	Kültz, 2005	cs Citrate synthase, mitochondrial (*Thunnus obesus*)	0,17901739	0,11636408	0,34942572
DnaJ/HSP40	Stress	Kültz, 2005	DNAJA1 DnaJ homolog subfamily A member 1 (*Bos taurus*)	-0,0357413	0,71843904	0,87019995
DnaJ/HSP40	Stress	Kültz, 2005	DNAJA2 DnaJ homolog subfamily A member 2 (*Bos taurus*)	0,18150822	0,07017261	0,2650064
DnaJ/HSP40	Stress	Kültz, 2005	DNAJA2 DnaJ homolog subfamily A member 2 (*Bos taurus*)	0,04247645	0,60364992	0,80765127
DnaJ/HSP40	Stress	Kültz, 2005	DNAJA3 DnaJ homolog subfamily A member 3, mitochondrial (*Homo sapiens*)	0,06949067	0,31794881	0,58841395
DnaJ/HSP40	Stress	Kültz, 2005	Dnaja3 DnaJ homolog subfamily A member 3, mitochondrial (*Mus musculus*)	-0,0043983	0,94553563	0,97784473
DnaJ/HSP40	Stress	Kültz, 2005	Dnaja4 DnaJ homolog subfamily A member 4 (*Mus musculus*)	0,44972268	0,02314699	0,14302908
DnaJ/HSP40	Stress	Kültz, 2005	DNAJB1 DnaJ homolog subfamily B member 1 (*Bos taurus*)	-0,2479703	0,05783629	0,23653499
DnaJ/HSP40	Stress	Kültz, 2005	DNAJB1 DnaJ homolog subfamily B member 1 (*Bos taurus*)	-0,1639037	0,24544656	0,51735593
DnaJ/HSP40	Stress	Kültz, 2005	Dnajb11 DnaJ homolog subfamily B member 11 (*Mus musculus*)	0,2431193	0,2151039	0,48421306
DnaJ/HSP40	Stress	Kültz, 2005	DNAJB12 DnaJ homolog subfamily B member 12 (*Homo sapiens*)	0,02549595	0,85990797	0,93826281
DnaJ/HSP40	Stress	Kültz, 2005	Dnajb12 DnaJ homolog subfamily B member 12 (*Mus musculus*)	-0,134784	0,26770777	0,54082974

DnaJ/HSP40	Stress	Kültz, 2005	Dnajb14 DnaJ homolog subfamily B member 14 (*Mus musculus*)	0,00635806	0,9297732	0,97106798	
DnaJ/HSP40	Stress	Kültz, 2005	DNAJB4 DnaJ homolog subfamily B member 4 (*Bos taurus*)	-0,1594646	0,08142115	0,28783044	
DnaJ/HSP40	Stress	Kültz, 2005	Dnajb5 DnaJ homolog subfamily B member 5 (*Mus musculus*)	-0,0069921	0,95523642	0,98183108	
DnaJ/HSP40	Stress	Kültz, 2005	DNAJB6 DnaJ homolog subfamily B member 6 (*Macaca fascicularis*)	0,00145631	0,98694081	0,99501831	
DnaJ/HSP40	Stress	Kültz, 2005	dnajb6-b DnaJ homolog subfamily B member 6-B (*Xenopus laevis*)	0,61006077	0,0011255	0,02633305	*
DnaJ/HSP40	Stress	Kültz, 2005	DNAJB9 DnaJ homolog subfamily B member 9 (*Homo sapiens*)	0,1460925	0,22877974	0,50034814	
DnaJ/HSP40	Stress	Kültz, 2005	Dnajb13 DnaJ homolog subfamily B member 13 (*Mus musculus*)	0,13460262	0,5279619	NA	
DnaJ/HSP40	Stress	Kültz, 2005	Dnajb9 DnaJ homolog subfamily B member 9 (*Rattus norvegicus*)	-0,0628816	0,6748741	0,84927531	
DnaJ/HSP40	Stress	Kültz, 2005	Dnajc1 DnaJ homolog subfamily C member 1 (*Mus musculus*)	0,00585137	0,97450812	0,98975567	
DnaJ/HSP40	Stress	Kültz, 2005	dnajc10 DnaJ homolog subfamily C member 10 (*Xenopus laevis*)	0,10610631	0,39034194	0,6516064	
DnaJ/HSP40	Stress	Kültz, 2005	DNAJC11 DnaJ homolog subfamily C member 11 (*Homo sapiens*)	0,08536099	0,38471985	0,64692664	
DnaJ/HSP40	Stress	Kültz, 2005	Dnajc11 DnaJ homolog subfamily C member 11 (*Mus musculus*)	0,21920236	0,07662637	0,27862688	
DnaJ/HSP40	Stress	Kültz, 2005	DNAJC13 DnaJ homolog subfamily C member 13 (*Homo sapiens*)	-0,0483966	0,65775407	0,83848613	
DnaJ/HSP40	Stress	Kültz, 2005	Dnajc14 DnaJ homolog subfamily C member 14	-0,052349	0,62853723	0,82337224	

			(*Mus musculus*)			
DnaJ/HSP40	Stress	Kültz, 2005	DNAJC16 DnaJ homolog subfamily C member 16 (*Gallus gallus*)	-0,029718	0,70630494	0,86434373
DnaJ/HSP40	Stress	Kültz, 2005	DNAJC16 DnaJ homolog subfamily C member 16 (*Homo sapiens*)	0,03739141	0,65876462	0,83899293
DnaJ/HSP40	Stress	Kültz, 2005	DNAJC17 DnaJ homolog subfamily C member 17 (*Homo sapiens*)	0,1120517	0,2702668	0,54394755
DnaJ/HSP40	Stress	Kültz, 2005	DNAJC18 DnaJ homolog subfamily C member 18 (*Bos taurus*)	0,03481691	0,6672229	0,84444235
DnaJ/HSP40	Stress	Kültz, 2005	dnajc2 DnaJ homolog subfamily C member 2 (*Danio rerio*)	0,37292936	0,02894158	0,16243513
DnaJ/HSP40	Stress	Kültz, 2005	dnajc21 DnaJ homolog subfamily C member 21 (*Danio rerio*)	0,25408699	0,10812931	0,33538748
DnaJ/HSP40	Stress	Kültz, 2005	dnajc22 DnaJ homolog subfamily C member 22 (*Xenopus tropicalis*)	-0,2994548	0,048528	0,21606727
DnaJ/HSP40	Stress	Kültz, 2005	DNAJC24 DnaJ homolog subfamily C member 24 (*Homo sapiens*)	0,02187165	0,88806147	0,95089903
DnaJ/HSP40	Stress	Kültz, 2005	dnajc25 DnaJ homolog subfamily C member 25 (*Xenopus tropicalis*)	0,23227578	0,17185702	0,42934732
DnaJ/HSP40	Stress	Kültz, 2005	dnajc27 DnaJ homolog subfamily C member 27 (*Danio rerio*)	-0,1849614	0,01809832	0,12456891
DnaJ/HSP40	Stress	Kültz, 2005	DNAJC3 DnaJ homolog subfamily C member 3 (*Homo sapiens*)	0,2276701	0,05541614	0,2312985
DnaJ/HSP40	Stress	Kültz, 2005	DNAJC30 DnaJ homolog subfamily C member 30 (*Homo sapiens*)	-0,1752619	0,10543791	0,33064352
DnaJ/HSP40	Stress	Kültz, 2005	DNAJC30 DnaJ homolog subfamily C member 30 (*Homo sapiens*)	0,25257003	0,23328839	0,50494743

DnaJ/HSP40	Stress	Kültz, 2005	Dnajc4 DnaJ homolog subfamily C member 4 (*Mus musculus*)	0,2751197	0,00702109	0,07344488	
DnaJ/HSP40	Stress	Kültz, 2005	Dnajc5 DnaJ homolog subfamily C member 5 (*Rattus norvegicus*)	0,07265772	0,5117175	0,74747086	
DnaJ/HSP40	Stress	Kültz, 2005	dnajc5 DnaJ homolog subfamily C member 5 (*Torpedo californica*)	0,4114178	0,00539704	0,06240544	
DnaJ/HSP40	Stress	Kültz, 2005	dnajc5 DnaJ homolog subfamily C member 5 (*Torpedo californica*)	-0,2375847	0,20527141	0,47204225	
DnaJ/HSP40	Stress	Kültz, 2005	dnajc5 DnaJ homolog subfamily C member 5 (*Xenopus laevis*)	0,14448282	0,02209352	0,13920085	
DnaJ/HSP40	Stress	Kültz, 2005	DNAJC6 Putative tyrosine-protein phosphatase auxilin (*Bos taurus*)	0,01673022	0,8637702	0,93995569	
DnaJ/HSP40	Stress	Kültz, 2005	DNAJC8 DnaJ homolog subfamily C member 8 (*Homo sapiens*)	0,1616395	0,06614509	0,25662629	
DnaJ/HSP40	Stress	Kültz, 2005	Dnajc9 DnaJ homolog subfamily C member 9 (*Mus musculus*)	0,53052605	0,00115683	0,02674162	*
Enolase (glycolysis)	Stress	Kültz, 2005	eno4 Enolase-like protein ENO4 (*Danio rerio*)	0,14440751	0,50943685	NA	
Enolase (glycolysis)	Stress	Kültz, 2005	ENO1 Alpha-enolase (*Gallus gallus*)	0,2826528	0,0178996	0,12386554	
Enolase (glycolysis)	Stress	Kültz, 2005	ENO2 Gamma-enolase (*Homo sapiens*)	0,14702294	0,03028003	0,16626849	
Enolase (glycolysis)	Stress	Kültz, 2005	ENO2 Gamma-enolase (*Homo sapiens*)	0,04704805	0,65764808	0,83848613	
Enolase (glycolysis)	Stress	Kültz, 2005	ENO3 Beta-enolase (*Salmo salar*)	0,50747962	0,00045652	0,01580124	*
FtsH/proteasome-regulatory subunit	Stress	Kültz, 2005	Yme1l1 ATP-dependent zinc metalloprotease YME1L1 (*Mus musculus*)	0,36519981	0,00715933	0,07424671	
FtsH/proteasome-regulatory subunit	Stress	Kültz, 2005	Yme1l1 ATP-dependent zinc metalloprotease YME1L1 (*Rattus norvegicus*)	-0,1728105	0,09635154	0,31473423	

Glutathione reductase	Stress	Kültz, 2005	Gsr Glutathione reductase, mitochondrial (*Mus musculus*)	-0,1045629	0,10245657	0,32597357	
Glycerol-3-phosphate dehydrogenase	Stress	Kültz, 2005	gpd1 Glycerol-3-phosphate dehydrogenase [NAD(+)], cytoplasmic (*Takifugu rubripes*)	0,84857478	2,3651E-07	8,1306E-05	*
Glycerol-3-phosphate dehydrogenase	Stress	Kültz, 2005	gpd1 Glycerol-3-phosphate dehydrogenase [NAD(+)], cytoplasmic (*Takifugu rubripes*)	-0,289349	0,07302165	0,27108681	
Glycerol-3-phosphate dehydrogenase	Stress	Kültz, 2005	gpd1l Glycerol-3-phosphate dehydrogenase 1-like protein (*Danio rerio*)	0,3146587	0,05072033	0,22143051	
Glycerol-3-phosphate dehydrogenase	Stress	Kültz, 2005	gpd1l Glycerol-3-phosphate dehydrogenase 1-like protein (*Xenopus tropicalis*)	0,07080786	0,51509394	0,74950492	
Glycerol-3-phosphate dehydrogenase	Stress	Kültz, 2005	GPD2 Glycerol-3-phosphate dehydrogenase, mitochondrial (*Homo sapiens*)	-0,1643592	0,2797175	0,55213205	
GrpE	Stress	Kültz, 2005	GRPEL1 GrpE protein homolog 1, mitochondrial (*Bos taurus*)	0,06325665	0,53424353	0,76214896	
Hsp 47	Stress	Abdel-Gawad & Khalil, 2013	SERPINH1 Serpin H1 (*Gallus gallus*)	0,72302959	1,4035E-05	0,00170173	*
Hsp 47	Stress	Abdel-Gawad & Khalil, 2013	SERPINH1 Serpin H1 (*Gallus gallus*)	-0,0936289	0,30818958	0,57860825	
HSP60	Stress	Kültz, 2005	HSPD1 60 kDa heat shock protein, mitochondrial (*Gallus gallus*)	0,32446635	0,08534515	0,29511123	
HSP70	stress	Kültz, 2005	Heat shock 70 kDa protein 1 (*Oryzias latipes*)	0,75200875	0,00043952	0,0154065	*
HSP70	stress	Kültz, 2005	Heat shock 70 kDa protein 1 (*Oryzias latipes*)	0,51146259	0,01921057	0,12848486	
HSP70	stress	Kültz, 2005	Heat shock 70 kDa protein 1 (*Oryzias latipes*)	-0,2190849	0,2116296	0,48045302	
HSP70	stress	Kültz, 2005	Heat shock 70 kDa protein 1 (*Oryzias latipes*)	0,19838258	0,3485764	0,61651539	
HSP70	stress	Kültz, 2005	Heat shock 70 kDa protein 1 (*Oryzias latipes*)	-0,0440985	0,80789728	0,91452048	
Hydroxyacylglutathione hydrolase	Stress	Kültz, 2005	hagh Hydroxyacylglutathione hydrolase, mitochondrial (*Danio rerio*)	0,02300864	0,8347145	0,92593186	

Inositol monophosphataseb	Stress	Kültz, 2005	IMPA1 Inositol monophosphatase 1 (*Bos taurus*)	0,05184595	0,74797155	0,88554062
Inositol monophosphataseb	Stress	Kültz, 2005	IMPA1 Inositol monophosphatase 1 (*Homo sapiens*)	0,18523506	0,11343719	0,34470888
Inositol monophosphataseb	Stress	Kültz, 2005	impad1 Inositol monophosphatase 3 (*Danio rerio*)	0,12119763	0,34025252	0,60959877
Isocitrate dehydrogenase	Stress	Kültz, 2005	IDH1 Isocitrate dehydrogenase [NADP] cytoplasmic (*Bos taurus*)	0,28400279	0,10216866	0,32573221
Isocitrate dehydrogenase	Stress	Kültz, 2005	IDH2 Isocitrate dehydrogenase [NADP], mitochondrial (*Bos taurus*)	0,09239702	0,6126502	0,81285845
Isocitrate dehydrogenase	Stress	Kültz, 2005	IDH2 Isocitrate dehydrogenase [NADP], mitochondrial (Fragment) (*Sus scrofa*)	0,41259168	0,02310097	0,14288211
Isocitrate dehydrogenase	Stress	Kültz, 2005	Idh3a Isocitrate dehydrogenase [NAD] subunit alpha, mitochondrial (*Mus musculus*)	0,22716399	0,03054447	0,16701441
Isocitrate dehydrogenase	Stress	Kültz, 2005	Idh3a Isocitrate dehydrogenase [NAD] subunit alpha, mitochondrial (*Mus musculus*)	0,21198584	0,11574912	0,34829518
Isocitrate dehydrogenase	Stress	Kültz, 2005	IDH3B Isocitrate dehydrogenase [NAD] subunit beta, mitochondrial (*Macaca fascicularis*)	0,20792493	0,07551283	0,27619271
Isocitrate dehydrogenase	Stress	Kültz, 2005	Idh3g Isocitrate dehydrogenase [NAD] subunit gamma 1, mitochondrial (*Mus musculus*)	0,09609688	0,13468964	0,37731496
Isocitrate dehydrogenase	Stress	Kültz, 2005	IDH3G Isocitrate dehydrogenase [NAD] subunit gamma, mitochondrial (*Bos taurus*)	0,24112538	0,00857829	0,08256659
Lon protease/protease La	Stress	Kültz, 2005	Lonp1 Lon protease homolog, mitochondrial (*Rattus norvegicus*)	0,23151518	0,07473851	0,27475204
Lon protease/protease La	Stress	Kültz, 2005	lonp2 Lon protease homolog 2, peroxisomal (*Danio rerio*)	-0,1610909	0,14909542	0,39905742
Long-chain fatty acid ABC transporter	Stress	Kültz, 2005	SLC27A1 Long-chain fatty acid transport protein 1 (*Homo sapiens*)	0,22987098	0,04734361	0,21305296

Long-chain fatty acid ABC transporter	Stress	Kültz, 2005	SLC27A4 Long-chain fatty acid transport protein 4 (*Homo sapiens*)	-0,302294	0,03880116	0,19024607	
Long-chain fatty acid ABC transporter	Stress	Kültz, 2005	SLC27A6 Long-chain fatty acid transport protein 6 (*Homo sapiens*)	-0,2187064	0,03966388	0,19274368	
Long-chain fatty acid ABC transporter	Stress	Kültz, 2005	SLC27A6 Long-chain fatty acid transport protein 6 (*Homo sapiens*)	0,10965643	0,33765059	0,60784251	
Long-chain-fatty-acid CoA ligase	Stress	Kültz, 2005	ACSBG2 Long-chain-fatty-acid--CoA ligase ACSBG2 (*Gallus gallus*)	0,26324513	0,01828256	0,12507792	
Long-chain-fatty-acid CoA ligase	Stress	Kültz, 2005	acsbg2 Long-chain-fatty-acid--CoA ligase ACSBG2 (*Xenopus laevis*)	-0,1774469	0,3071425	0,57747223	
Long-chain-fatty-acid CoA ligase	Stress	Kültz, 2005	ACSL1 Long-chain-fatty-acid--CoA ligase 1 (*Cavia porcellus*)	0,24714912	0,11213524	0,342445	
Long-chain-fatty-acid CoA ligase	Stress	Kültz, 2005	ACSL1 Long-chain-fatty-acid--CoA ligase 1 (*Cavia porcellus*)	-0,137843	0,12322295	0,35977816	
Long-chain-fatty-acid CoA ligase	Stress	Kültz, 2005	Acsl1 Long-chain-fatty-acid--CoA ligase 1 (*Rattus norvegicus*)	0,24586071	0,09865126	0,31905681	
Long-chain-fatty-acid CoA ligase	Stress	Kültz, 2005	ACSL3 Long-chain-fatty-acid--CoA ligase 3 (*Homo sapiens*)	0,24283601	0,08858378	0,30141313	
Long-chain-fatty-acid CoA ligase	Stress	Kültz, 2005	ACSL3 Long-chain-fatty-acid--CoA ligase 3 (*Homo sapiens*)	0,22061061	0,11238775	0,34294451	
Long-chain-fatty-acid CoA ligase	Stress	Kültz, 2005	Acsl5 Long-chain-fatty-acid--CoA ligase 5 (*Rattus norvegicus*)	-0,1850102	0,14385297	0,39110723	
Long-chain-fatty-acid CoA ligase	Stress	Kültz, 2005	Acsl6 Long-chain-fatty-acid--CoA ligase 6 (*Rattus norvegicus*)	0,49570693	0,00098853	0,02456686	*
MsrA/PMSR	Stress	Kültz, 2005	MSRA Mitochondrial peptide methionine sulfoxide reductase (*Homo sapiens*)	-0,0915487	0,28799847	0,55982612	
MutL/MLH	Stress	Kültz, 2005	Mlh1 DNA mismatch repair protein Mlh1 (*Mus	0,04082803	0,64902527	0,83361481	

			musculus)				
MutS/MSH	Stress	Kültz, 2005	MSH4 MutS protein homolog 4 (*Homo sapiens*)	0	1	NA	
MutS/MSH	Stress	Kültz, 2005	MSH5 MutS protein homolog 5 (*Homo sapiens*)	-0,1970061	0,34243713	NA	
MutS/MSH	Stress	Kültz, 2005	MSH5 MutS protein homolog 5 (*Homo sapiens*)	0,03753203	0,7546332	NA	
MutS/MSH	Stress	Kültz, 2005	MSH2 DNA mismatch repair protein Msh2 (*Chlorocebus aethiops*)	0,51151545	0,00377669	0,05088112	
MutS/MSH	Stress	Kültz, 2005	MSH3 DNA mismatch repair protein Msh3 (*Homo sapiens*)	-0,0995816	0,16765154	0,42405589	
MutS/MSH	Stress	Kültz, 2005	MSH4 MutS protein homolog 4 (*Homo sapiens*)	0,38453742	0,00064716	0,01906865	*
Nucleoside diphosphate kinase	Stress	Kültz, 2005	NME1-2 Nucleoside diphosphate kinase A 2 (*Bos taurus*)	0,59847051	2,2563E-05	0,00232397	*
Nucleoside diphosphate kinase	Stress	Kültz, 2005	NME2 Nucleoside diphosphate kinase B (*Sus scrofa*)	0,2197657	0,05337044	0,22736444	
Nucleoside diphosphate kinase	Stress	Kültz, 2005	Nme4 Nucleoside diphosphate kinase, mitochondrial (*Mus musculus*)	-0,0970111	0,50750571	0,74496106	
Nucleoside diphosphate kinase	Stress	Kültz, 2005	NME5 Nucleoside diphosphate kinase homolog 5 (*Homo sapiens*)	-0,0996001	0,56199909	0,78106877	
Nucleoside diphosphate kinase	Stress	Kültz, 2005	nme6 Nucleoside diphosphate kinase 6 (*Danio rerio*)	0,34354491	0,0176642	0,1228972	
Nucleoside diphosphate kinase	Stress	Kültz, 2005	Nme7 Nucleoside diphosphate kinase 7 (*Mus musculus*)	-0,0870626	0,38070009	0,64347861	
Peroxiredoxin	Stress	Kültz, 2005	Peroxiredoxin (*Cynops pyrrhogaster*)	0,28317077	0,04578585	0,20928344	
Peroxiredoxin	Stress	Kültz, 2005	PRDX1 Peroxiredoxin-1 (*Myotis lucifugus*)	0,10261875	0,50591659	0,74416442	
Peroxiredoxin	Stress	Kültz, 2005	PRDX5 Peroxiredoxin-5, mitochondrial (*Homo sapiens*)	0,27819123	0,03948861	0,19217809	

Peroxiredoxin	Stress	Kültz, 2005	PRDX6 Peroxiredoxin-6 (*Gallus gallus*)	0,11702803	0,37874032	0,64157233	
Peroxiredoxin	Stress	Kültz, 2005	PRDX6 Peroxiredoxin-6 (*Gallus gallus*)	0,12437383	0,52598911	0,75686903	
Petidyl-prolyl isomerase	Stress	Kültz, 2005	PIN1 Peptidyl-prolyl cis-trans isomerase NIMA-interacting 1 (*Homo sapiens*)	0,06150119	0,45205853	0,70167428	
Phosphoglucomutase	Stress	Kültz, 2005	PGM1 Phosphoglucomutase-1 (*Macaca fascicularis*)	0,4641161	0,02358025	0,14505539	
Phosphoglucomutase	Stress	Kültz, 2005	Pgm1 Phosphoglucomutase-1 (*Mus musculus*)	0,41084113	0,00744629	0,0760366	
Phosphoglucomutase	Stress	Kültz, 2005	PGM2 Phosphoglucomutase-2 (*Homo sapiens*)	-0,1766138	0,03182217	0,17084366	
Proline oxidase	Stress	Kültz, 2005	PRODH Proline dehydrogenase 1, mitochondrial (*Bos taurus*)	-0,2054665	0,19667464	0,46188242	
Proline oxidase	Stress	Kültz, 2005	Prodh Proline dehydrogenase 1, mitochondrial (*Mus musculus*)	0,2348219	0,21772139	0,48739556	
Proline oxidase	Stress	Kültz, 2005	prodh2 Probable proline dehydrogenase 2 (*Xenopus laevis*)	0,02217484	0,90136449	0,95641945	
Proline oxidase	Stress	Kültz, 2005	prodh2 Probable proline dehydrogenase 2 (*Xenopus laevis*)	0,01047238	0,91384906	0,96273749	
Protease II/prolyl endopetidase	Stress	Kültz, 2005	FAP Prolyl endopeptidase FAP (*Bos taurus*)	0,51949238	0,01403818	0,10899804	
Protease II/prolyl endopetidase	Stress	Kültz, 2005	Prep Prolyl endopeptidase (*Mus musculus*)	0,34268662	0,00210075	0,03675176	*
Putative oxidoreductase YIM4	Stress	Kültz, 2005	glyr1 Putative oxidoreductase GLYR1 (*Danio rerio*)	-0,0486269	0,53659593	0,76352741	
Quinone oxidoreductase	Stress	Kültz, 2005	MT-ND1 NADH-ubiquinone oxidoreductase chain 1 (*Gadus morhua*)	0,22931012	0,02899064	0,16249258	
Quinone oxidoreductase	Stress	Kültz, 2005	NDUFS1 NADH-ubiquinone oxidoreductase 75 kDa subunit, mitochondrial (*Homo sapiens*)	-0,4328279	0,02529622	0,15093629	
Quinone oxidoreductase	Stress	Kültz, 2005	NDUFS1 NADH-ubiquinone oxidoreductase 75	0,10646753	0,3734363	0,63740402	

			kDa subunit, mitochondrial (*Pongo pygmaeus*)			
Quinone oxidoreductase	Stress	Kültz, 2005	TP53I3 Quinone oxidoreductase PIG3 (*Homo sapiens*)	0,21148297	0,17237532	0,43013509
RecA/Rad51	Stress	Kültz, 2005	RAD51 DNA repair protein RAD51 homolog 1 (*Homo sapiens*)	0,59041721	0,00675341	0,07213101
RecA/Rad51	Stress	Kültz, 2005	RAD51B DNA repair protein RAD51 homolog 2 (*Homo sapiens*)	0,01210745	0,91109003	0,96114389
RecA/Rad51	Stress	Kültz, 2005	Rad51c DNA repair protein RAD51 homolog 3 (*Mus musculus*)	0,26207945	0,13633057	0,38013967
RecA/Rad51	Stress	Kültz, 2005	Rad51d DNA repair protein RAD51 homolog 4 (*Mus musculus*)	-0,1042373	0,26461028	0,53697381
Ribosomal RNA methyltransferase	Stress	Kültz, 2005	Emg1 Ribosomal RNA small subunit methyltransferase NEP1 (*Mus musculus*)	0,28353456	0,0360367	0,1825891
Ribosomal RNA methyltransferase	Stress	Kültz, 2005	Ftsj2 rRNA methyltransferase 2, mitochondrial (*Mus musculus*)	0,2641028	0,05587136	0,2322432
Ribosomal RNA methyltransferase	Stress	Kültz, 2005	Nop2 Probable 28S rRNA (cytosine-C(5))-methyltransferase (*Mus musculus*)	0,20879802	0,16526881	0,42078939
Ribosomal RNA methyltransferase	Stress	Kültz, 2005	nsun4 5-methylcytosine rRNA methyltransferase NSUN4 (*Xenopus laevis*)	0,29807049	0,03100567	0,16857255
Ribosomal RNA methyltransferase	Stress	Kültz, 2005	NSUN5 Probable 28S rRNA (cytosine-C(5))-methyltransferase (*Homo sapiens*)	0,1810732	0,03156768	0,17028353
Ribosomal RNA methyltransferase	Stress	Kültz, 2005	RNMTL1 rRNA methyltransferase 3, mitochondrial (*Homo sapiens*)	0,19448019	0,04187058	0,19926315
Ribosomal RNA methyltransferase	Stress	Kültz, 2005	Wbscr22 Probable 18S rRNA (guanine-N(7))-methyltransferase (*Mus musculus*)	0,1145616	0,28732639	0,55936237
SelB	Stress	Kültz, 2005	EEFSEC Selenocysteine-specific elongation factor (*Homo sapiens*)	0,15193804	0,21309646	0,48202354

Serine protease	Stress	Kültz, 2005	F56F10.1 Putative serine protease F56F10.1 (*Caenorhabditis elegans*)	-0,2348553	0,20272516	0,46896309	
Serine protease	Stress	Kültz, 2005	Htra1 Serine protease HTRA1 (*Rattus norvegicus*)	0,27358533	0,20814437	0,47607253	
Serine protease	Stress	Kültz, 2005	htra1a Serine protease HTRA1A (*Danio rerio*)	0,49455987	3,2877E-05	0,00309192	*
Serine protease	Stress	Kültz, 2005	htra1a Serine protease HTRA1A (*Danio rerio*)	0,26790745	0,14365531	0,3907442	
Serine protease	Stress	Kültz, 2005	HTRA2 Serine protease HTRA2, mitochondrial (*Bos taurus*)	0,03796473	0,58841845	0,79770662	
Serine protease	Stress	Kültz, 2005	HTRA3 Serine protease HTRA3 (*Homo sapiens*)	0,62953631	0,0013168	0,02878293	*
Serine protease	Stress	Kültz, 2005	Htra3 Serine protease HTRA3 *(Rattus norvegicus*)	0,0573185	0,53513815	0,76254132	
Serine protease	Stress	Kültz, 2005	Prss16 Thymus-specific serine protease (*Mus musculus*)	-0,1445216	0,40008456	0,66015322	
Serine protease	Stress	Kültz, 2005	Prss23 Serine protease 23 (*Rattus norvegicus*)	0,25774794	0,20917566	0,47752487	
Serine protease	Stress	Kültz, 2005	PRSS27 Serine protease 27 (*Homo sapiens*)	-0,3783159	0,05058709	0,22114934	
Serine protease	Stress	Kültz, 2005	PRSS27 Serine protease 27 (*Homo sapiens*)	-0,2235663	0,31336512	0,58417651	
Serine protease	Stress	Kültz, 2005	PRSS27 Serine protease 27 (*Homo sapiens*)	-0,1689149	0,35045715	0,61835298	
Serine protease	Stress	Kültz, 2005	Prss27 Serine protease 27 (*Rattus norvegicus*)	-0,4477272	0,01058158	0,09291189	
Serine protease	Stress	Kültz, 2005	Prss27 Serine protease 27 (*Rattus norvegicus*)	-0,3882908	0,02738613	0,15679384	
Serine protease	Stress	Kültz, 2005	Prss27 Serine protease 27 (*Rattus norvegicus*)	-0,1124598	0,28200636	0,55447837	
Serine protease	Stress	Kültz, 2005	htra1b Serine protease HTRA1B (*Danio rerio*)	0,33267545	0,13864094	NA	
Serine protease	Stress	Kültz, 2005	Prss57 Serine protease 57 (*Mus musculus*)	-0,0636853	0,77297281	NA	
Serine protease	Stress	Kültz, 2005	PRSS35 Inactive serine protease 35 (*Homo*	0,34689785	0,08343624	0,29165477	

			sapiens)			
Succinate-semialdehyde dehydrogenase	Stress	Kültz, 2005	Aldh5a1 Succinate-semialdehyde dehydrogenase, mitochondrial (*Rattus norvegicus*)	0,28853965	0,1201571	0,35523826
Superoxide dismutase	Stress	Kültz, 2005	SOD2 Superoxide dismutase [Mn], mitochondrial (*Bos taurus*)	0,11683525	0,08559427	0,29567674
Superoxide dismutase	Stress	Kültz, 2005	SOD3 Extracellular superoxide dismutase [Cu-Zn] (*Homo sapiens*)	0,07964429	0,70538519	0,8639253
Thioredoxin	Stress	Kültz, 2005	Txnl1 Thioredoxin-like protein 1 (*Mus musculus*)	0,04447477	0,78270101	0,90273778
Thioredoxin	Stress	Kültz, 2005	TXNRD1 Thioredoxin reductase 1, cytoplasmic (*Sus scrofa*)	-0,0512619	0,75458975	0,88933462
Thioredoxin	Stress	Kültz, 2005	Txnrd2 Thioredoxin reductase 2, mitochondrial (*Mus musculus*)	-0,0142916	0,86703013	0,94149238
Thioredoxin	Stress	Kültz, 2005	TXNRD3 Thioredoxin reductase 3 (*Homo sapiens*)	-0,0301976	0,72375005	0,87277156
Topoisomerase I/III	Stress	Kültz, 2005	TOP1 DNA topoisomerase 1 (*Chlorocebus aethiops*)	0,05236843	0,78277204	0,90273778
Topoisomerase I/III	Stress	Kültz, 2005	TOP1 DNA topoisomerase 1 (*Homo sapiens*)	0,24329612	0,06667777	0,25778282
Topoisomerase I/III	Stress	Kültz, 2005	top1 DNA topoisomerase 1 (*Xenopus laevis*)	0,10503409	0,16905713	0,42582499
Topoisomerase I/III	Stress	Kültz, 2005	TOP3B DNA topoisomerase 3-beta-1 (*Homo sapiens*)	0,16576543	0,21566015	0,48509066

Table 8 Supplementary information and References to Figure 4

Reference Nr in Figure 4	Species	Stressor	time	tissue	mRNA/ Protein	Reference	HSP70	
1	*Solea senegalensis*	pCO_2	1 month	pooled whole larvae	Protein	Pimentel et al., 2015	1.4849861	
1	*Solea senegalensis*	pCO_2	1 month	pooled whole larvae	Protein	Pimentel et al., 2015	2.1364074	*
2	*Carassius carassius*	hypoxia	7 day	brain	mRNA	Stenslokken et al., 2010	0.9610716	
2	*Carassius carassius*	hypoxia	7 day	heart	mRNA	Stenslokken et al., 2010	1.6777697	*
2	*Carassius carassius*	hypoxia	7 day	brain	mRNA	Stenslokken et al., 2010	11.9058795	*
3	*Paralichthys olivaceus*	salinity	2 weeks	liver	mRNA	Choi, 2010	29.3750244	*
3	*Paralichthys olivaceus*	salinity	2 weeks	liver	mRNA	Choi, 2010	53.3750634	*
3	*Paralichthys olivaceus*	salinity	2 weeks	liver	mRNA	Choi, 2010	55.8750634	*
4	*Mylio macrocepahlus*	salinity	8 month	liver	Protein	Deane et al., 2002	1.8479852	*
4	*Mylio macrocepahlus*	salinity	8 month	liver	Protein	Deane et al., 2002	0.4994684	
4	*Mylio macrocepahlus*	salinity	8 month	liver	Protein	Deane et al., 2002	1.6354201	*
5	*Sparus sarba*	salinity	1 month	liver	mRNA	Deane and Woo, 2004	0.9412418	
5	*Sparus sarba*	salinity	1 month	liver	mRNA	Deane and Woo, 2004	1.1841484	
5	*Sparus sarba*	salinity	1 month	liver	mRNA	Deane and Woo, 2004	1.0051117	
5	*Sparus sarba*	salinity	1 month	kidney	mRNA	Deane and Woo, 2004	1.0950025	
5	*Sparus sarba*	salinity	1 month	kidney	mRNA	Deane and Woo, 2004	0.9226692	
5	*Sparus sarba*	salinity	1 month	kidney	mRNA	Deane and Woo, 2004	0.8055263	
5	*Sparus sarba*	salinity	1 month	gill	mRNA	Deane and Woo, 2004	1.6199978	
5	*Sparus sarba*	salinity	1 month	gill	mRNA	Deane and Woo, 2004	0.3400002	*
5	*Sparus sarba*	salinity	1 month	gill	mRNA	Deane and Woo, 2004	0.3266668	*
7	*Paralichthys olivaceus*	salinity	48h	whole larvae	mRNA	Wu et al., 2017	1.3661964	
7	*Paralichthys olivaceus*	salinity	48h	whole larvae	mRNA	Wu et al., 2017	1.5563394	
3	*Paralichthys olivaceus*	temperature	2 weeks	liver	mRNA	Choi, 2010	49.6489394	*
3	*Paralichthys olivaceus*	temperature	2 weeks	liver	mRNA	Choi, 2010	46.3822024	*
1	*Solea senegalensis*	temperature	1 month	pooled whole	Protein	Pimentel et al., 2015	1.0679184	
1	*Solea senegalensis*	temperature	1 month	pooled whole	Protein	Pimentel et al., 2015	1.7344358	*
8	*Melanotaenia duboulayi*	temperature	14 days	liver	mRNA	Smith et al., 2013	9.9000000	*
This study	*Gadus morhua*	pCO_2	36 days	whole larvae	mRNA		1.6800000	*

References

Choi, C.Y., 2010. Environmental stress-related gene expression and blood physiological responses in olive flounder (*Paralichthys olivaceus*) exposed to osmotic and thermal stress. Animal Cells Syst. 14, 17–23. doi:10.1080/19768351003764940

Deane, E.E., Kelly, S.P., Luk, J.C.Y., Woo, N.Y.S., 2002. Chronic salinity adaptation modulates hepatic heat shock protein and insulin-like growth factor I expression in black sea bream. Mar. Biotechnol. 4, 193–205. doi:10.1007/s10126-001-0091-5

Deane, E.E., Woo, N.Y.S., 2004. Differential gene expression associated with euryhalinity in sea bream (*Sparus sarba*). Am. J. Physiol. Regul. Integr. Comp. Physiol. 287, 1054–1063. doi:10.1152/ajpregu.00347.2004.

Pimentel, M.S., Faleiro, F., Diniz, M., Machado, J., Pousão-Ferreira, P., Peck, M.A., Pörtner, H.O., Rosa, R., 2015. Oxidative stress and digestive enzyme activity of flatfish larvae in a changing ocean. PLoS One 10, 1–18. doi:10.1371/journal.pone.0134082

Smith, S., Bernatchez, L., Beheregaray, L.B., 2013. RNA-seq analysis reveals extensive transcriptional plasticity to temperature stress in a freshwater fish species. BMC Genomics 14, 1. doi:10.1186/1471-2164-14-375

Stenslokken, K.O., Ellefsen, S., Larsen, H.K., Vaage, J., Nilsson, G., E., 2010. Expression of heat shock proteins in anoxic crucian carp (*Carassius carassius*): support for cold as a preparatory cue for anoxia. AJP Regul. Integr. Comp. Physiol. 298, R1499–R1508. doi:10.1152/ajpregu.00675.2009

Wu, H., Liu, J., Lu, Z., Xu, L., Ji, C., Wang, Q., Zhao, J., 2017. Metabolite and gene expression responses in juvenile flounder *Paralichthys olivaceus* exposed to reduced salinities. Fish Shellfish Immunol. 63, 417–423. doi:10.1016/j.fsi.2017.02.042
